# Organization of primate amygdalar–thalamic pathways for emotions

**DOI:** 10.1371/journal.pbio.3000639

**Published:** 2020-02-27

**Authors:** Clare Timbie, Miguel Á. García-Cabezas, Basilis Zikopoulos, Helen Barbas

**Affiliations:** 1 Department of Anatomy and Neurobiology, Boston University School of Medicine, Boston, Massachusetts, United States of America; 2 Neural Systems Lab, Department of Health Sciences, Boston University, Boston, Massachusetts, United States of America; 3 Human Systems Neuroscience Lab, Department of Health Sciences, Boston University, Boston, Massachusetts, United States of America; Darmouth College, UNITED STATES

## Abstract

Studies on the thalamus have mostly focused on sensory relay nuclei, but the organization of pathways associated with emotions is not well understood. We addressed this issue by testing the hypothesis that the primate amygdala acts, in part, like a sensory structure for the affective import of stimuli and conveys this information to the mediodorsal thalamic nucleus, magnocellular part (MDmc). We found that primate sensory cortices innervate amygdalar sites that project to the MDmc, which projects to the orbitofrontal cortex. As in sensory thalamic systems, large amygdalar terminals innervated excitatory relay and inhibitory neurons in the MDmc that facilitate faithful transmission to the cortex. The amygdala, however, uniquely innervated a few MDmc neurons by surrounding and isolating large segments of their proximal dendrites, as revealed by three-dimensional high-resolution reconstruction. Physiologic studies have shown that large axon terminals are found in pathways issued from motor systems that innervate other brain centers to help distinguish self-initiated from other movements. By analogy, the amygdalar pathway to the MDmc may convey signals forwarded to the orbitofrontal cortex to monitor and update the status of the environment in processes deranged in schizophrenia, resulting in attribution of thoughts and actions to external sources.

## Abstract 2

Pathways from the amygdala target specific neurons in the thalamic magnocellular mediodorsal nucleus to enable speedy and faithful transmission of emotional information to prefrontal cortex.

## Introduction

The amygdala has a key role in sensing the significance of stimuli and events, but the organization of pathways that enable this vital function is not well understood. Input from the external and internal environments is an essential component for the sensory-related function of the amygdala. As such, the primate amygdala receives a wealth of projections from all high-order sensory association and polymodal cortices that represent the external environment, as well as from limbic cortices that signal the status of the internal milieu [1–5]. The amygdala sends a strong projection to the mediodorsal thalamic nucleus, magnocellular part (MDmc) [6,7], which projects to several prefrontal areas but has as its main target the posterior orbitofrontal cortex (pOFC) [8,9].

We had hypothesized that the projection from sensory and polymodal areas to the amygdala may be part of a feedforward stream to the thalamus and cortex that conveys information about the emotional import of a stimulus, a scene, or an event [7,10,11]. The serial pathways that connect the amygdala with the pOFC through the thalamus are reminiscent of sensory pathways that connect peripheral sensory receptors with sensory thalamic nuclei, which then project to the cortex. In the sensory systems, pathways from the periphery are robust and have a distinct organization within the thalamic sensory relay nuclei. Specifically, pathways that carry signals about the features of the environment participate in classical synaptic triads, whereby a sensory axon terminal forms a synapse with a dendrite of a thalamic relay neuron as well as with an inhibitory neuron, which is presynaptic to the relay neuron (reviewed in [12]). The triadic arrangement thus transmits signals from the periphery to both excitatory relay and thalamic inhibitory neurons in a pattern that is essential for faithful transmission of thalamic signals to the cortex [13]. We reasoned that if the amygdala functions as a “sensory organ” for salient signals with affective significance, it may be similarly organized into triads in the MDmc, which then projects to the pOFC.

The pathway connecting the amygdala with the MDmc remains largely unexplored at the synaptic level in primates, and even its organization at the level of the system is not well understood. We addressed both issues from the system to the synapse for the amygdalar projection to the MDmc and compared it to the classical triads described for the pathway from the retina to the dorsal lateral geniculate nucleus (LGN) in rhesus monkeys. In addition, in spite of the classical description of triads in the thalamus (e.g., [12,14,15]), to the best of our knowledge, such a structure has never been visualized in three dimensions in any species, an analysis that can provide insights on the organization of the primate thalamus.

Our findings reveal that the sequence of sensory-related pathways to the amygdala and then to the MDmc has strong parallels with pathways in the sensory systems. Beyond the similarities, however, we found novel, to our knowledge, complex, and unusually strong synaptic interactions of amygdalar pathways in the MDmc that appear to be unique to this system. These findings attest to the significance of the amygdalar pathways for the synthesis of cognitive and emotional processes for action.

## Results

### Sequential pathways link high-order sensory association areas with the amygdala and the MDmc

**Fig 1:**
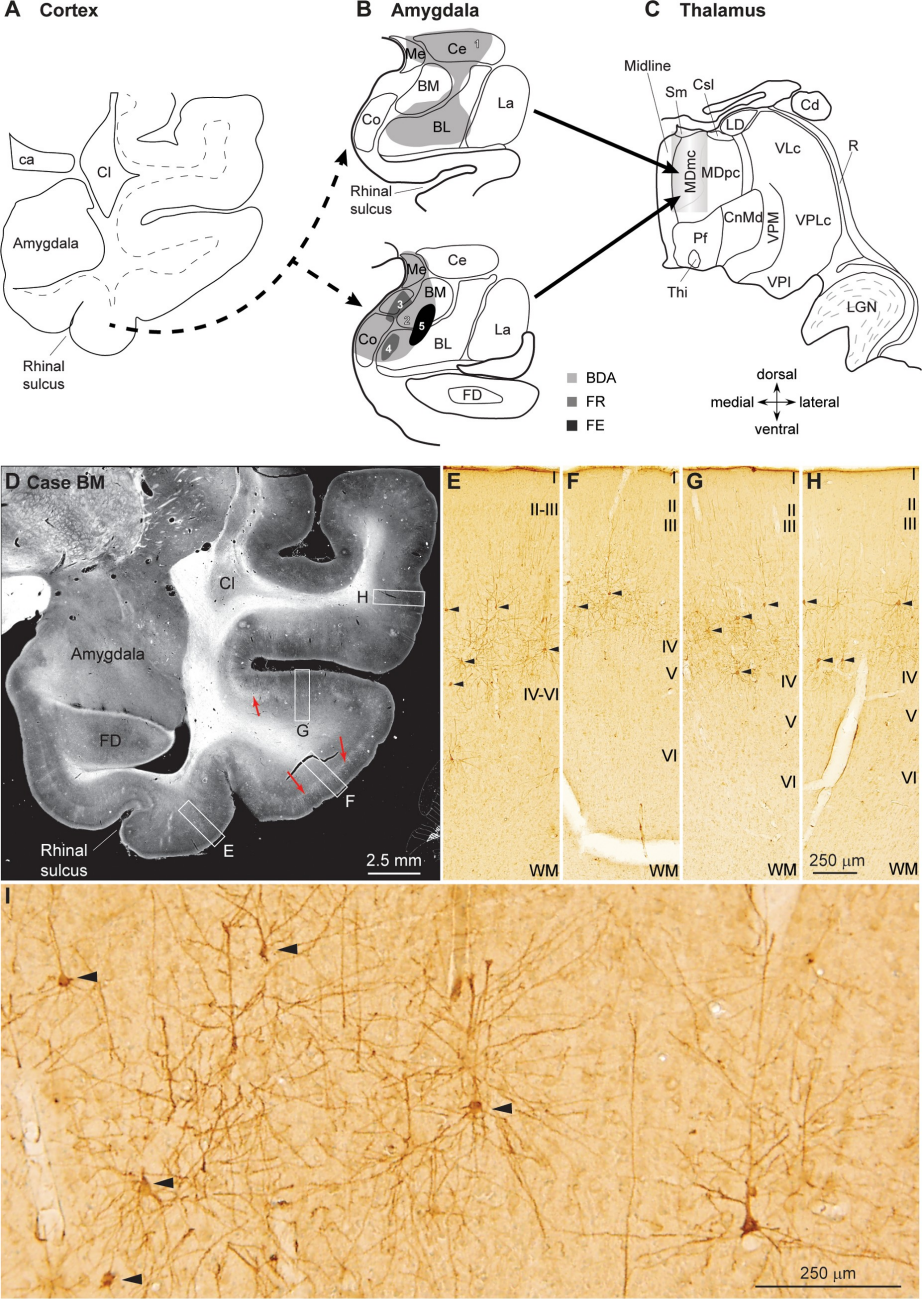
Experimental design to label sequential pathways from sensory association cortices to the amygdala and from the amygdala to the MDmc. (A) A diagram of coronal section through the temporal cortex shows the origin of retrogradely-labeled projection neurons in temporal sensory association cortices that project to the amygdala (dashed arrows); (B) diagrams of coronal sections show injection of bidirectional tracers in the amygdala. (C) Sketch of coronal section shows termination of pathways from the amygdala in the MDmc, anterogradely labeled from the same injection sites (solid arrows). (D) Low-magnification darkfield photomicrograph of coronal section through the temporal lobe shows neurons and terminals (white in cortex, highlighted by red arrows) in rhinal polymodal (E) and inferior temporal visual (F, G) and auditory association cortices (H) labeled after injection of a bidirectional tracer in the amygdala (4 in B). (E–H) Brightfield photomicrographs of insets from D show that labeled projection neurons (arrowheads) are found mostly in layer III, revealing “feedforward” mode of projection from sensory association areas to the amygdala. (I) High magnification of perirhinal region from D that includes the area shown in inset E, with labeled neurons and axon terminals. Labeled projection neurons marked by arrowheads are the ones shown in E. 1–5 designate individual cases: 1, case BB; 2, case BD; 3, case BL; 4, case BM; 5, case BN. Roman numerals indicate cortical layers. Calibration bar in H applies to E–H. BDA, biotinylated dextran amine; BL, basolateral nucleus; BM, basomedial nucleus (also known as accessory basal); Ca, anterior commissure; Cd, caudate nucleus; Ce, central nucleus; Cl, claustrum; CnMd, centromedian nucleus; Co, cortical nucleus; Csl, central superior lateral nucleus; FD, fascia dentata; FE, Fluoro-emerald; FR, Fluoro-ruby; La, lateral nucleus; LD, lateral dorsal nucleus; LGN, lateral geniculate nucleus; MDmc, mediodorsal thalamic nucleus, magnocellular part; MDpc, mediodorsal thalamic nucleus, parvicellular part; Me, medial nucleus; Midline, midline nuclei; Pf, parafascicular nucleus; R, thalamic reticular nucleus; Sm, stria medullaris; Thi, habenulointerpeduncular tract; VLc, ventral lateral nucleus, caudal part; VPI, ventral posterior inferior nucleus; VPLc, ventral posterior lateral nucleus, caudal part; VPM, ventral medial nucleus; WM, white matter.

We first investigated whether there is continuity in pathways from sensory association cortices to the amygdala and then to the thalamic MDmc, as shown in Fig 1A–1C. We mapped these pathways after injection of bidirectional tracers in the amygdala (cases listed in Table 1). We found retrogradely-labeled neurons that project to the amygdala in polymodal cortices in the lateral rhinal region, in adjacent visual association cortices in the inferior temporal cortex, and in superior temporal auditory association cortices (Fig 1D). The predominant origin of these projections was layer III (Fig 1E–1I), confirming and extending our previous findings [11]. The upper layer projection from sensory association cortices to the amygdala is akin to the pattern seen in feedforward pathways that link earlier-processing with later-processing sensory areas in cortex [16]. From the same injection sites in the amygdala, we traced an anterograde pathway of labeled axons that terminated in the thalamic MDmc, consistent with previous findings [7,17]. This evidence attests to the continuity of the pathway from the amygdala to the MDmc. Taken together, these feedforward sequential pathways are consistent with the hypothesis that the amygdala can act as a sensor of the environment and conveys this information to the MDmc [7]. At the level of the system, the sequential pathways are thus akin to the pattern seen in the pathways from the sensory periphery to the thalamic relay nuclei. By extension, the pathways through the amygdala suggest sequential processing and elaboration of signals pertaining to the affective significance of stimuli and events.

**Table 1:** Rhesus monkey data and type of tracing analysis.

Case	Sex	Tracer Injected (Bidirectional)	Tracing Analysis				EM (in LGN)
			Cortex (retrograde)	Thalamus (anterograde)			
				LM (axon diameter in MDmc and midline)	LM (maps in MDmc)	EM (in MDmc)	
BB	F	BDA	+	+	+	−	−
BD	M	BDA	+	+	+	−	−
BL	M	FR	+	+	+	+	−
BN	M	FE	+	+	+	+	+
BM	F	FR	+	−	+	−	−

**Abbreviations:** BDA, biotinylated dextran amine; EM, electron microscopy; F, female; FE, Fluoro-emerald; FR, Fluoro-ruby; LGN, lateral geniculate nucleus; LM, light microscopy; M, male; MDmc, mediodorsal thalamic nucleus, magnocellular part; midline, midline thalamic nuclei; +, cases used for each analysis. Left column lists individual animal cases.

### Organizing features of the pathway from amygdala to the MDmc

**Fig 2:**
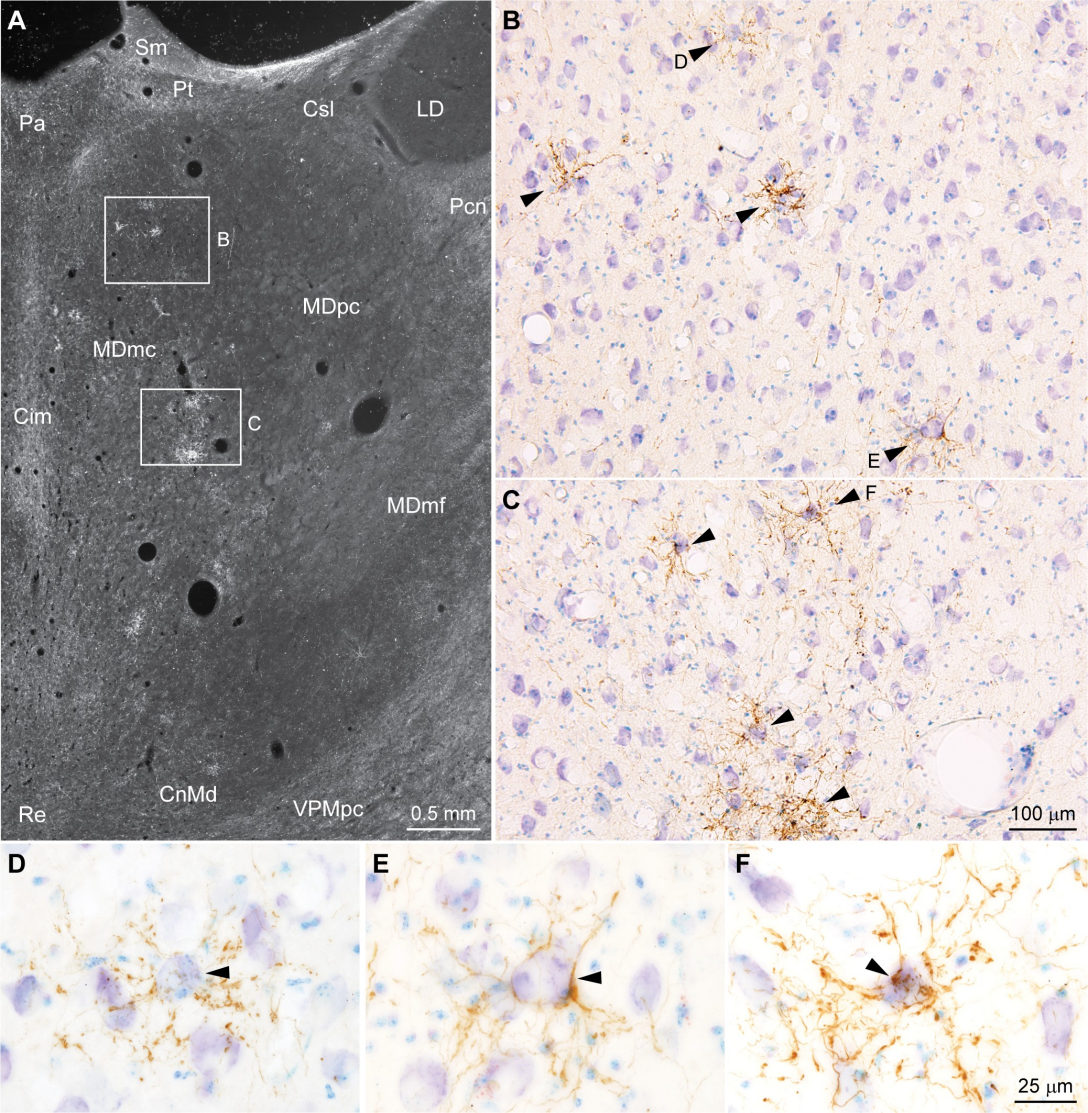
Pathways from the amygdala innervate the MDmc. (A) Low-power darkfield photomicrograph of coronal section through the MDmc shows focal clusters of terminations from amygdalar axons (white). (B and C) Brightfield photomicrographs of insets from A show distinct clusters of terminations from amygdalar axons (brown, arrowheads). (D–F) Stacks of high-power photomicrographs of the clusters in B and C through the entire thickness of the section show that the amygdalar axonal terminations (brown) are concentrated around bodies of MDmc neurons (arrowheads). Calibration bar in C applies to B and C. Calibration bar in F applies to D–F. Cim, central intermediate nucleus; CnMd, centromedian nucleus; Csl, central superior lateral nucleus; LD, lateral dorsal nucleus; MDmc, mediodorsal thalamic nucleus, magnocellular part; MDmf, mediodorsal thalamic nucleus, multiform part; MDpc, mediodorsal thalamic nucleus, parvicellular part; Pa, paraventricular nucleus; Pcn, paracentral nucleus; Pt, paratenial nucleus; Re, nucleus reuniens; Sm, stria medullaris; VPMpc, ventral posterior medial nucleus, parvicellular part.

**Fig 3:**
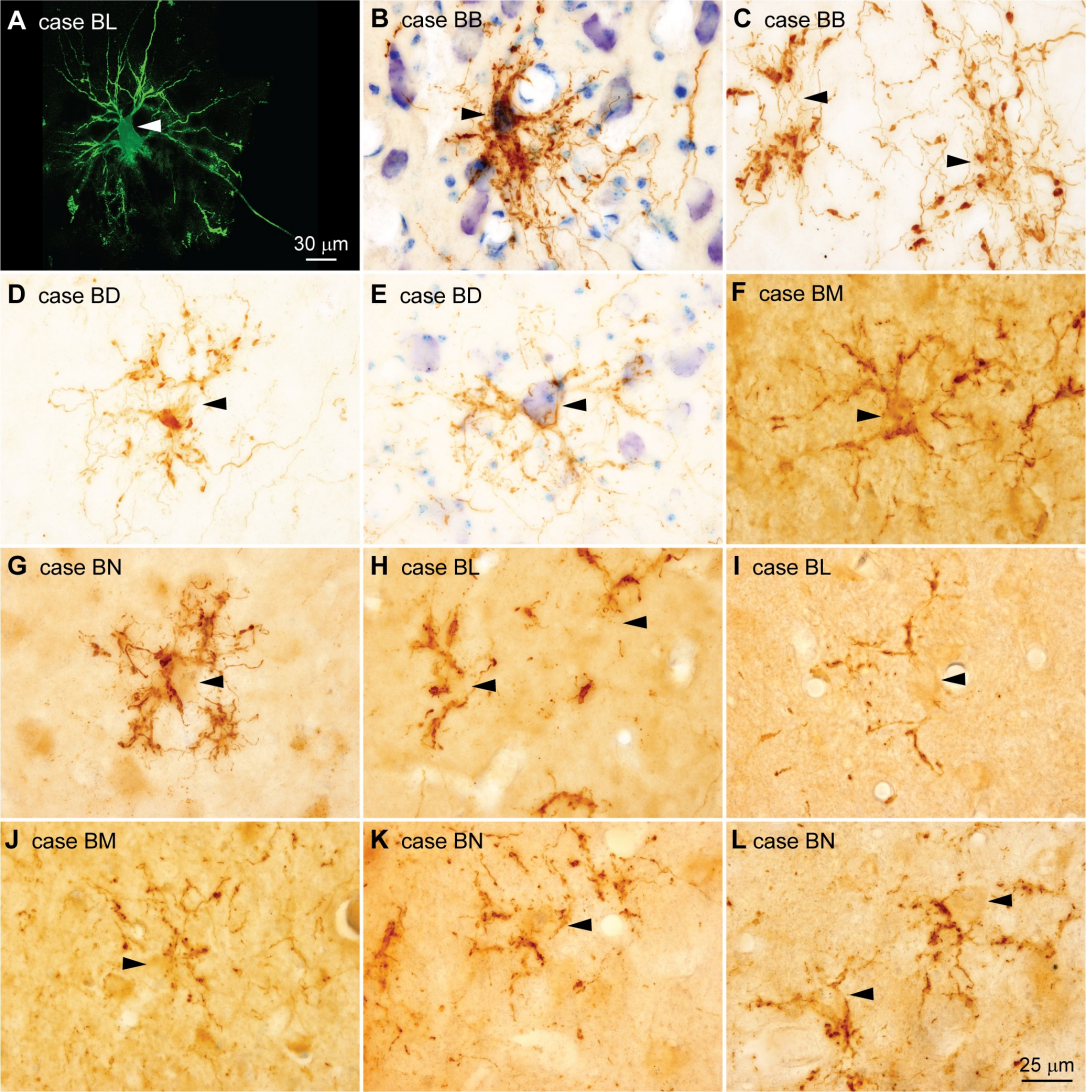
Pathways from the amygdala innervate the proximal dendrites of MDmc neurons. (A) Architecture of neurons in the MDmc. Photomicrograph shows multiple dendrites branching from the neuron body (arrowhead) in radial arrays. (B–G) Stacks of photomicrographs through the entire thickness of sections show that axons and terminals (brown) from the amygdala are arranged into complex radial structures centered around neuron bodies (arrowheads) and proximal dendrites. (H–L) In some cases, the radial arrangements of axons surround part of the dendritic arbor of MDmc neurons (arrowheads). Calibration bar in L applies to B–L. MDmc, mediodorsal thalamic nucleus, magnocellular part.

We then studied in detail the pathway from the amygdala to the MDmc from the system to the synapse. At the level of the system, we mapped the pathway using brightfield microscopy (*n* = 5 cases listed in Table 1). As shown in Fig 2A–2C, the pattern of termination had a striking periodicity (see also [6,18,19]). Amygdalar axons appeared to surround unlabeled neurons in MDmc (Fig 2B–2F, arrowheads), while most neighboring neurons appeared to be sparsely innervated or were untouched by the pathway. Labeled amygdalar axons framed MDmc cell bodies, suggesting innervation of proximal dendrites of the targeted neurons. The circular amygdalar axons around MDmc neurons gave rise to complex radial arrangements of axons forming octopus-like structures (Figs 2D–2F and 3). This pattern suggests innervation of proximal dendrites, which appear to extend to the radial dendritic tree of neurons we found to be characteristic of the MDmc (example seen in Fig 3A).

**Fig 4:**
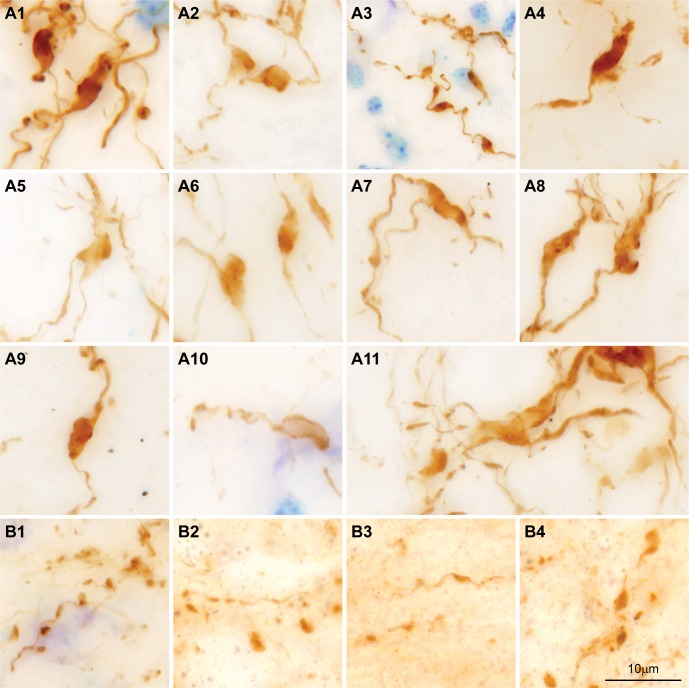
Comparison of amygdalar terminals in the MDmc and in nearby midline nuclei. (A1–A11) Stacks of photomicrographs through the entire thickness of sections show the complex structure of amygdalar terminals (brown) in the MDmc that include polymorphic enlargements of variable sizes along thin axons. (B1–B4) Stacks of photomicrographs through the entire thickness of sections in nearby midline nuclei show typical *en passant* and *terminaux* axon terminals (brown) from amygdalar pathways. Calibration bar in B4 applies to A1–A11 and B1–B4. MDmc, mediodorsal thalamic nucleus, magnocellular part.

At higher magnification, amygdalar axons included boutons of variable size. Numerous ovoid or asymmetric enlargements, presumed to be boutons, appeared along thin stretches of axons, which often branched and then fused, forming reticulated structures that resembled plant roots (Fig 4A1–4A11). The pathway from the amygdala to the MDmc appeared to be unidirectional because we saw no evidence of labeled neurons in the MDmc.

**Fig 5:**
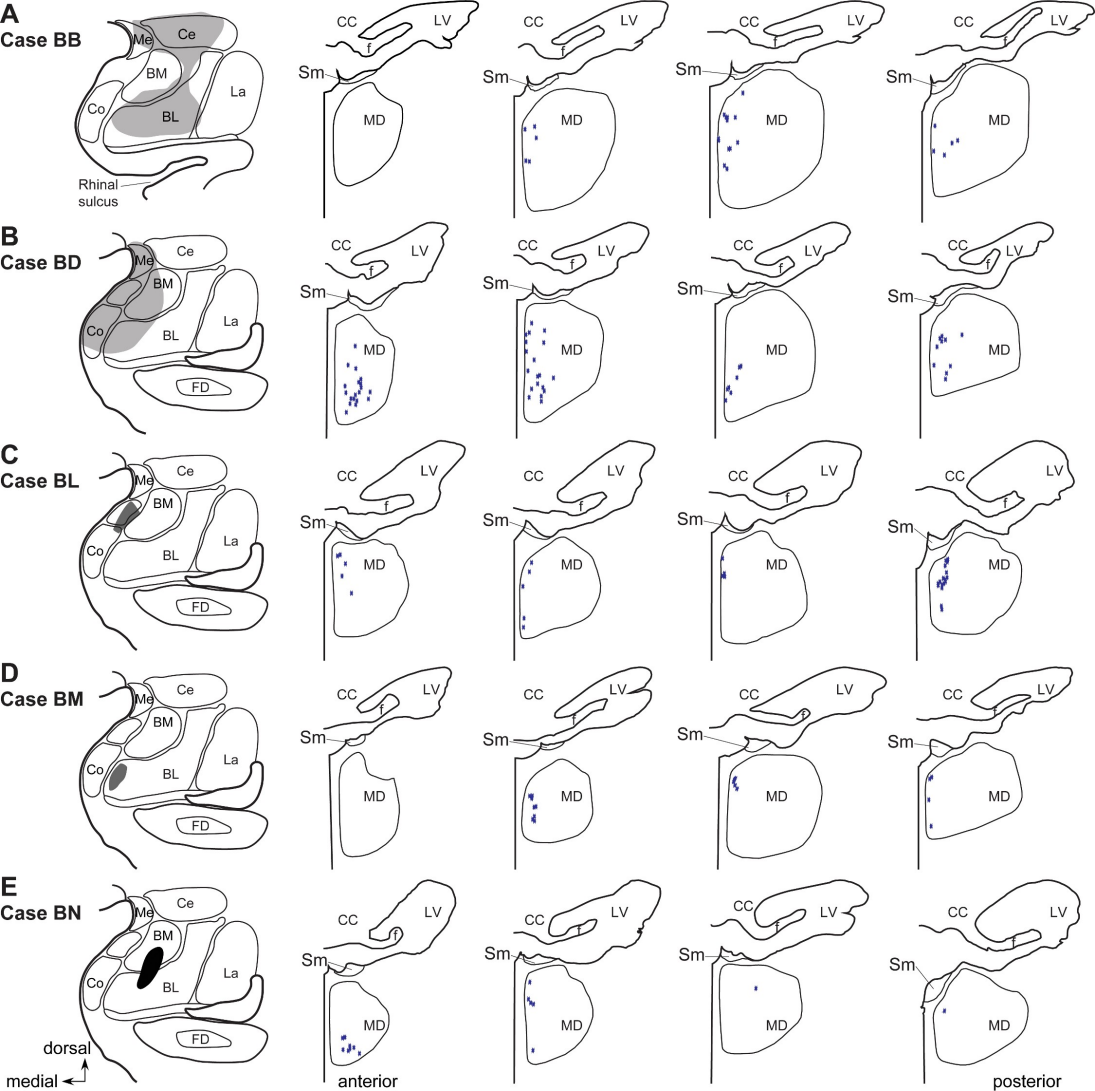
Amygdalar pathways innervate the anteroposterior extent of the MDmc. (A–E) Left panels show injection sites in the amygdala. Panels to the right show plots of the incidence of complex radially arranged axonal structures through anterior (left) to posterior (right) levels shown in coronal sections through the MDmc; each star represents one complex structure around an unlabeled neuron in the MDmc. Injections involving the cortical nuclei and the BM nuclei of the amygdala (B and C) show more of the complex axonal structures overall. BL, basolateral nucleus; BM, basomedial nucleus (also known as accessory basal); Ce, central nucleus; CC, corpus callosum; Co, cortical nucleus; f, fornix; FD, fascia dentata; La, lateral nucleus; LV, lateral ventricle; MD, mediodorsal thalamic nucleus; MDmc, mediodorsal thalamic nucleus, magnocellular part; Me, medial nucleus; Sm, stria medullaris.

We found the same focused innervation of single neurons in radial arrangements along the anteroposterior extent of the MDmc in all cases (Fig 5). Cases with the most medial injections in the amygdala that included the cortical nuclei and the basomedial nucleus (BM, also known as accessory basal) had more of these radial patterns of innervation (Fig 5B and 5C), but in all cases, the complex axon arrangements were restricted to the MDmc.

**Fig 6:**
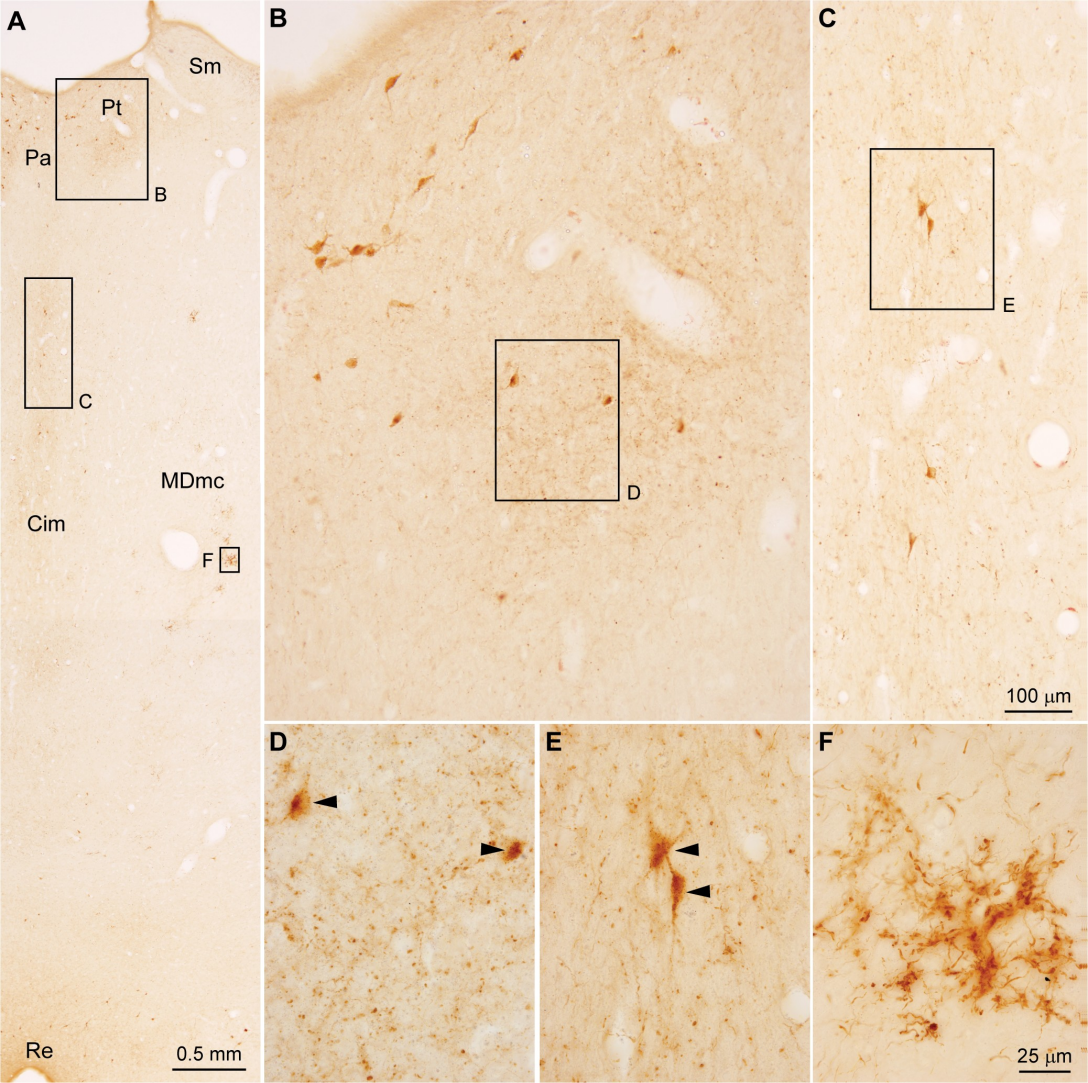
Amygdalar pathways to midline thalamic nuclei are diffuse and bidirectional. (A–C) Midline nuclei show diffuse amygdalar connections. (D and E) Some neurons in midline nuclei are labeled retrogradely (arrowheads) after injections in the amygdala. (F) In the adjacent MDmc, unidirectional amygdalar pathways terminate in complex radial arrangements, as seen in Fig 3. Calibration bar in C applies to B, C. Calibration bar in F applies to D–F. Cim, central intermediate nucleus; MDmc, mediodorsal thalamic nucleus, magnocellular part; Pa, paraventricular nucleus; Pt, paratenial nucleus; Re, nucleus reuniens; Sm, stria medullaris.

Amygdalar axons also innervated the midline nuclei in the same cases and sections, being densest in paraventricular (Pa), central intermediate (Cim), and reuniens nuclei (Fig 6A). However, the pattern of labeling in midline nuclei was diffuse (Fig 6B–6E), differing markedly from the punctuated and complex amygdalar terminations in the MDmc (Fig 6F). Furthermore, in contrast to the pleomorphic shapes and sizes of axonal bouton enlargements in the MDmc, terminals in midline nuclei were of typical *en passant* or *terminaux* types (Fig 4B1–4B4), as commonly seen in other pathways.

**Fig 7:**
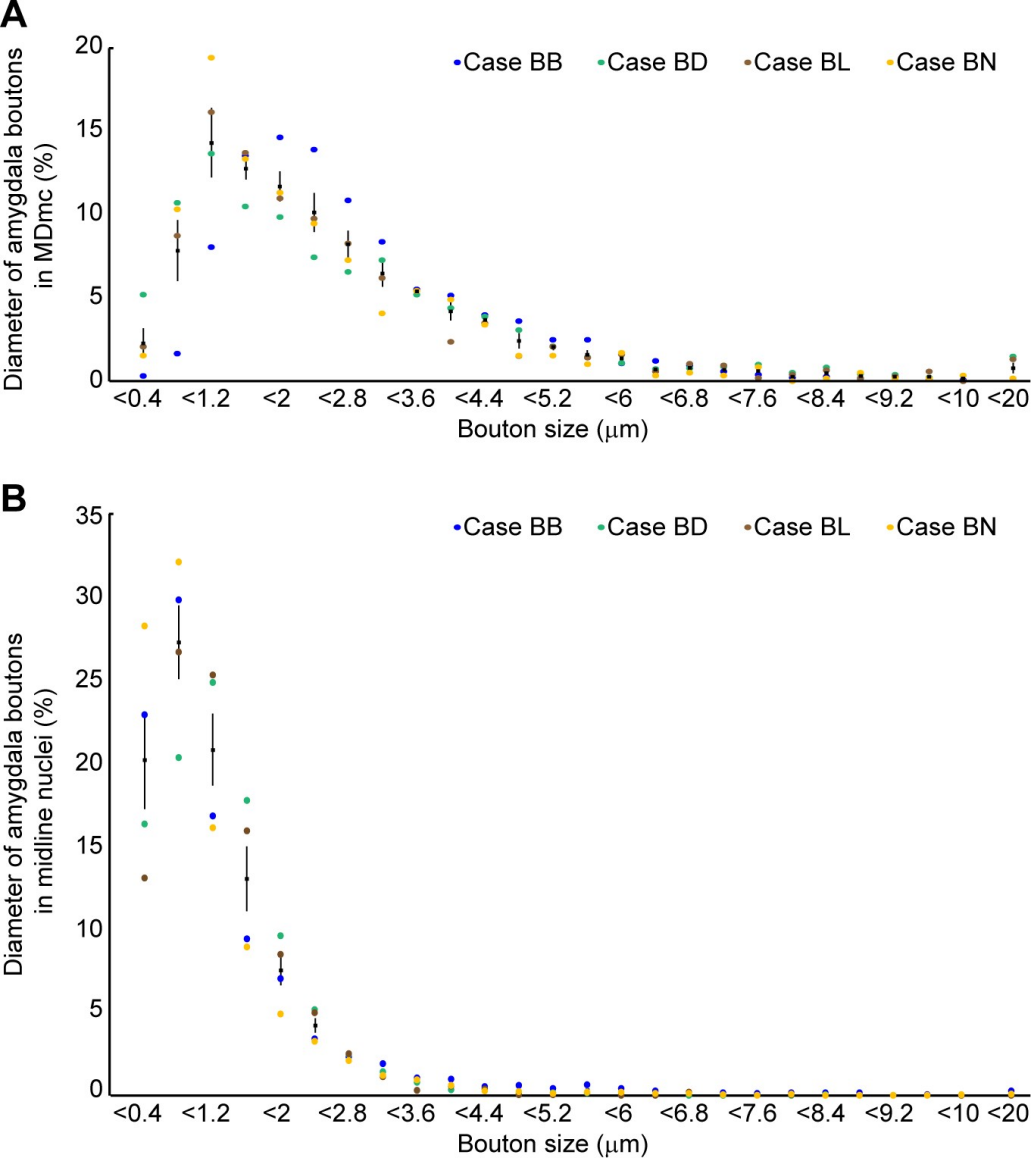
Amygdalar axon terminals are larger in the MDmc than in the midline thalamic nuclei. (A) Distribution of labeled boutons from amygdalar pathways in the MDmc plotted by size of the major diameter. (B) Distribution of labeled boutons from amygdalar pathways in midline nuclei plotted by size of the major diameter. Color dots show the mean frequency in 4 animals (cases BB, BD, BL, and BN). Black dots show the mean frequency across cases. Vertical lines on black dots show the standard error. The numerical data underlying this figure can be found in S1 Data. MDmc, mediodorsal thalamic nucleus, magnocellular part.

The amygdalar boutons in MDmc were also distinguished for their large size, being on average 2.5 times larger in their major diameter than boutons in the midline nuclei (average diameter: amygdalar boutons in MDmc: 2.54 μm ± 0.17, *n* = 5,369 from 4 cases listed in Table 1; in midline nuclei: 1.09 μm ± 0.10, *n* = 9,670, from the same cases; Fig 7). Unlike the pathway to the MDmc, connections with midline nuclei were bidirectional, evidenced by labeled neurons intermixed with labeled axons and terminals (Fig 6B–6E).

### Dendritic and axonal elements in the MDmc

**Fig 8:**
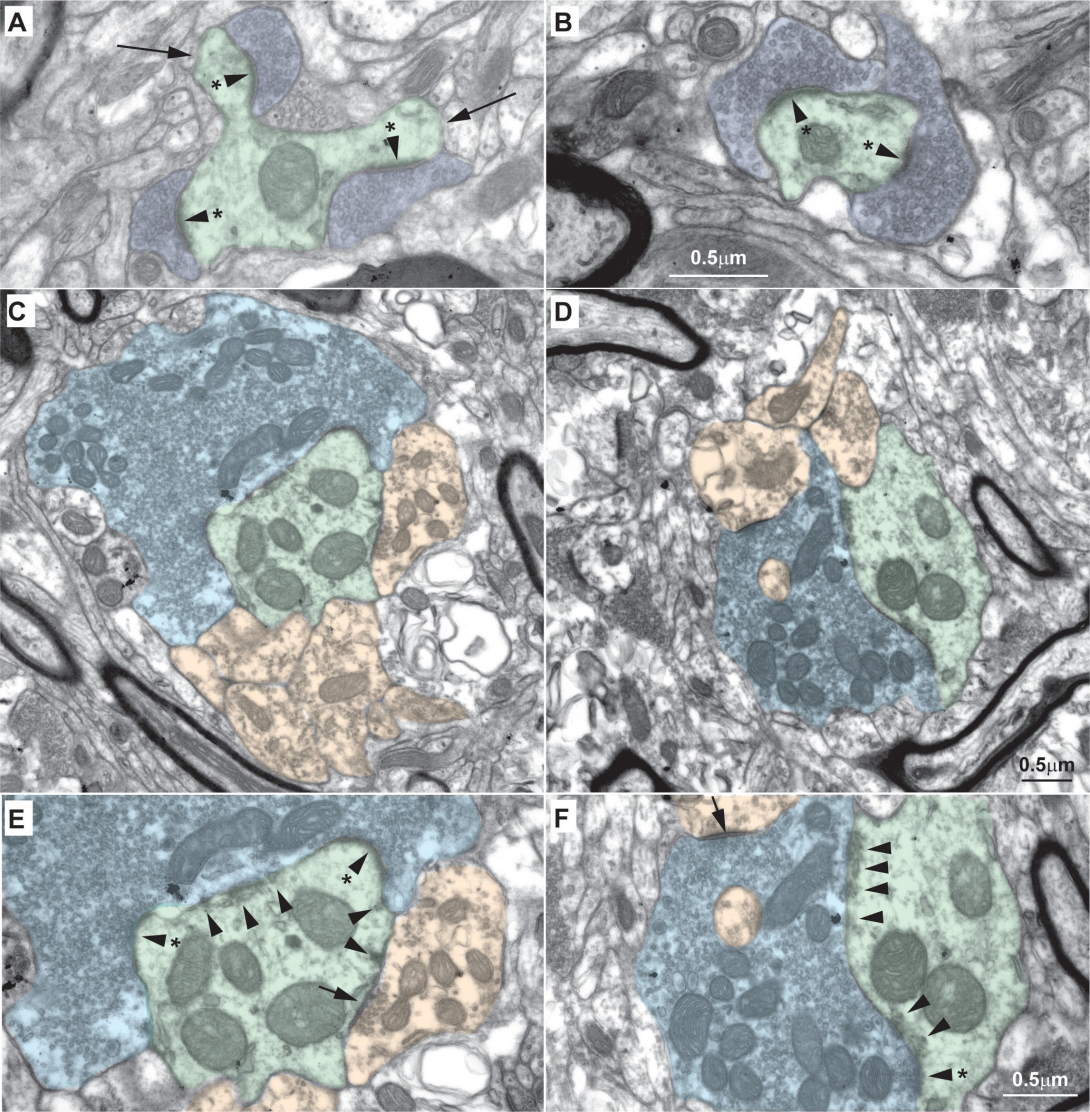
Fine structure of excitatory profiles in the MDmc of the macaque thalamus. (A, B) Some axon terminals in the MDmc are small, contain round vesicles, and form asymmetric (excitatory) synapses (arrowheads with asterisk, A and B). These round vesicle, small terminals (RSs; dark blue, A and B) form synapses with typical dendrites, some of which have cytoplasmic expansions (thorns, long arrows, A), that likely are part of thalamic relay neurons; we refer to these dendrites as EDs (green, A and F). (C–F) Other axon terminals in the MDmc are large, contain round vesicles, and form asymmetric (excitatory) synapses (arrowheads with asterisk, E and F). These large terminals with round vesicles, known as RLs (light blue, C–F) form synapses with EDs (green, C–F). RLs also form synapses with dendrites that have flat and pleomorphic vesicles and form symmetric (inhibitory) synapses (short arrow, E) with EDs. Dendrites with flat and pleomorphic vesicles are inhibitory (IDs; orange in C–F). RL terminals that form synapses with EDs also show many nonsynaptic contacts called puncta adherentia (arrowheads, E and F), a nonsynaptic adhesion; IDs also form some puncta adherentia with EDs (E). Calibration bar in B applies to A, B. Calibration bar in D applies to C, D. Calibration bar in F applies to E, F. ED, excitatory dendrite; ID, inhibitory dendrite; MDmc, mediodorsal thalamic nucleus, magnocellular part; RL, round vesicles, large terminal; RS, round vesicles, small terminal.

**Fig 9:**
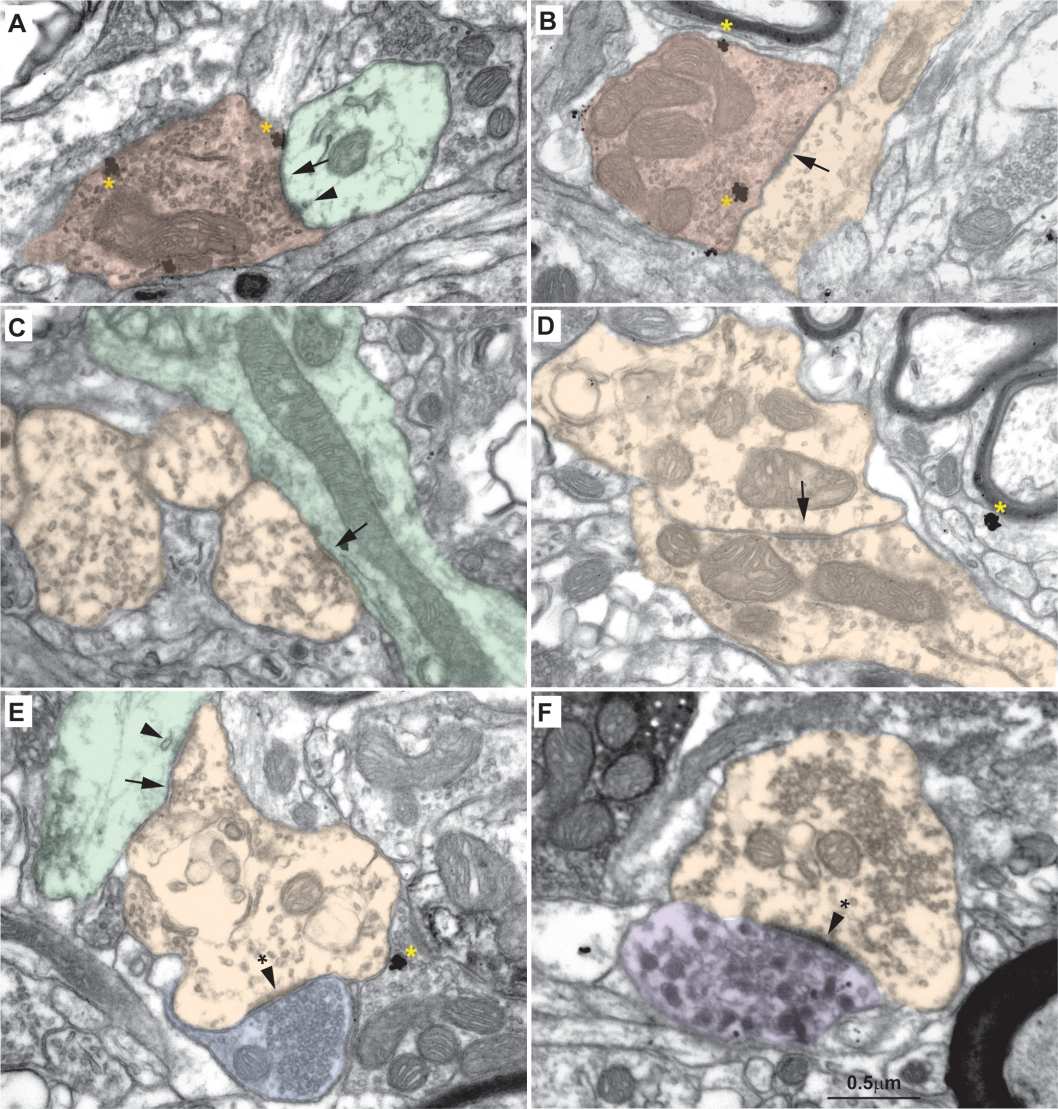
Fine structure of inhibitory profiles in the MDmc of the rhesus macaque thalamus. (A and B) Some axon terminals in the MDmc have dense cytoplasm and contain flat and pleomorphic vesicles. These inhibitory axon terminals (F, red, A and B) form symmetric (inhibitory) synapses (arrow) with EDs (green, A) and with IDs (orange, B); F terminals also form puncta adherentia (arrowhead, A) with EDs (green, A). (C, D) IDs (orange, B–F) contain a few flat and pleomorphic vesicles and can be differentiated from F terminals by their light cytoplasm, presence of microtubules, and relative paucity of vesicles. IDs form symmetric (inhibitory) synapses (arrows, C, E) with EDs (green, C, E) and other IDs (orange, D). (E) RSs also form asymmetric (excitatory) synapses (arrowhead with asterisk) with IDs (orange). The ID also forms puncta adherentia (arrowhead) with EDs (green, E). (F) We also identified one axon terminal that contained DCs and formed one asymmetric (excitatory) synapse (arrowhead with asterisk) with one ID. This profile is likely catecholaminergic, as described previously in the MD of rhesus monkeys [23]. Yellow asterisks show silver-enhanced gold labeling for PV. DC, dense-core vesicle; ED, excitatory dendrite; F, inhibitory axon terminal; ID, inhibitory dendrite; MD, mediodorsal thalamic nucleus; MDmc, mediodorsal thalamic nucleus, magnocellular part; PV, parvalbumin; RS, round vesicles, small terminal.

**Fig 10:**
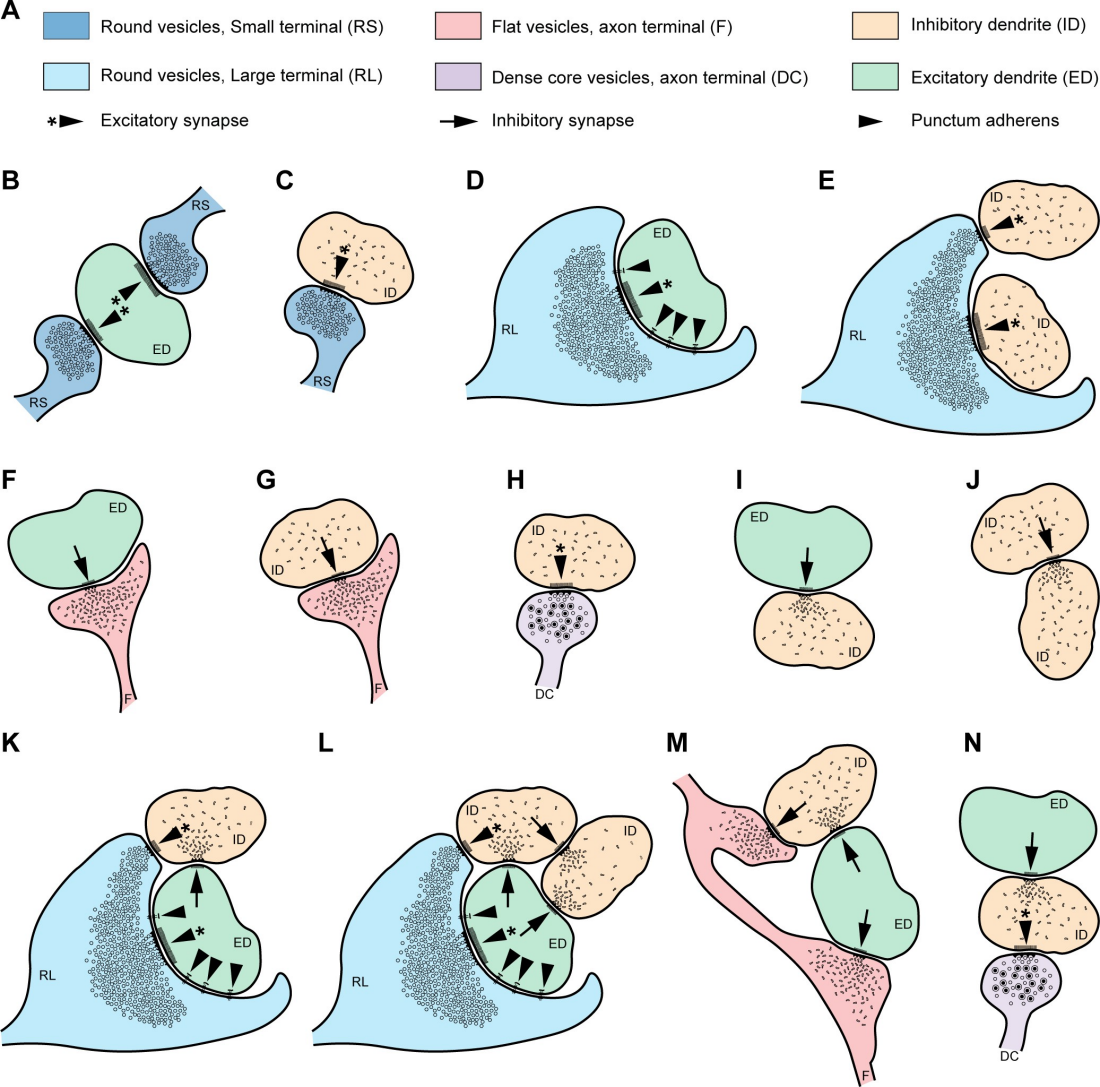
Summary of synaptic interactions in the MDmc of the rhesus macaque. (A) Summary of pre- and postsynaptic profiles and types of synaptic and nonsynaptic contacts between neurons found in the MDmc; color codes and abbreviations used are the same as in Figs 8 and 9. (B) RSs form excitatory synapses with EDs. (C) RSs also form excitatory synapses with IDs. (D) RLs form excitatory synapses and show several puncta adherentia (nonsynaptic structures) with EDs. (E) RLs also form excitatory synapses with IDs. (F, G) F terminals form inhibitory synapses with EDs (F) and IDs (G). (H) A terminal with DCs, which is likely catecholaminergic, forms an excitatory synapse with an ID. (I) An ID forms an inhibitory synapse with an ED. (J) An ID forms an inhibitory synapse with an ID. (K) The classical triadic arrangement consists of one RL that forms an excitatory synapse with one relay ED and another synapse on one ID; the same ID forms a synapse with the relay ED. (L) More complex synaptic arrangements consist of a classical triad (as in K) and one ID that inhibits both the ID and ED of the triad. (M) In inhibitory triads, described here for the first time, to our knowledge, 2 axon terminals of F type along the same axon form a synapse with one ED and another synapse on an ID; the same ID forms a synapse with the relay ED. (N) The axon terminal with DCs that we identified formed an excitatory synapse with one ID, which in turn formed an inhibitory synapse on one ED. DC, dense-core vesicle; ED, excitatory dendrite; F, inhibitory axon terminal; ID, inhibitory dendrite; MDmc, mediodorsal thalamic nucleus, magnocellular part; RL, round vesicles, large terminal; RS, round vesicles, small terminal.

To analyze the fine structure of the pathway, we first identified types of presynaptic and postsynaptic profiles in the neuropil of MDmc, conducted for the first time in a primate species at very high resolution. We used the terms that describe other parts of the thalamus in the literature, such as the lateral (parvicellular) part of the mediodorsal thalamic nucleus (MDpc) and the ventral anterior nuclei [20–24]. The types of profiles we found are described in Figs 8 and 9 and summarized in diagrams in Fig 10. Briefly, we identified the same profiles as in sensory relay [25] and other nuclei, including the main constituents of synaptic triads: presence of one round large (RL) terminal, named for its size and content of round vesicles, which formed an asymmetric (excitatory) synapse with one relay excitatory dendrite (ED) and one inhibitory dendrite (ID) containing sparser vesicles, which in turn formed a synapse on the same relay ED (Fig 10K). RL terminals also formed multiple puncta adherentia, a nonsynaptic adhesion, with their postsynaptic relay ED (Fig 8E and 8F; arrowheads). The other type of terminal seen had flattened, pleomorphic vesicles (F terminal), characteristic of inhibitory endings, which belong to either local inhibitory neurons or to the thalamic reticular nucleus or ventral pallidum. Details of the neuropil analysis are described and illustrated in Figs 8–10.

### Synaptic features of amygdalar boutons in the MDmc: 2D analysis

In the context of the neuropil composition, we then conducted 2D analysis using EM to investigate amygdalar axon terminal boutons in the MDmc. Amygdalar axons had large boutons in the MDmc (mean diameter ± SE, 3.4 ± 0.28 μm, range 1.1 μm to 7.0 μm, *n* = 38 boutons from 2 cases, listed in Table 1), which exceeded in size amygdalar boutons that innervate the pOFC directly, as described [7].

**Fig 11:**
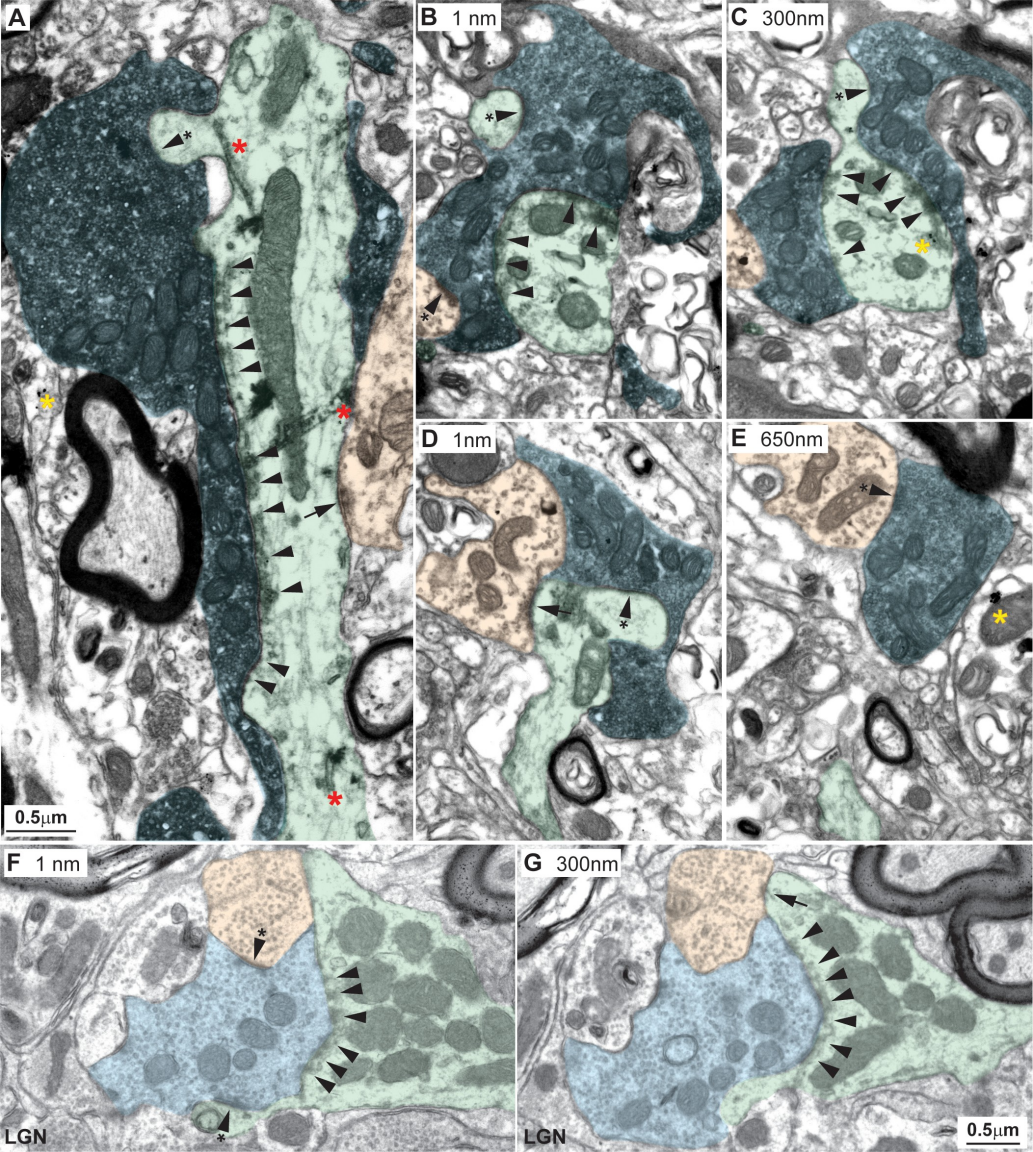
Amygdalar pathways in the MDmc form synaptic triads. (A–C) Labeled amygdalar terminals (black label with blue overlay) were large and contained round vesicles, RLs. Amygdalar terminals were also attached to EDs (green) through many puncta adherentia (A–C, arrowheads), which together covered a significant part of the dendritic surface. Synapses of amygdalar terminals with EDs were found mostly on thorns (A–C, arrowhead with asterisk on green shading). EDs innervated by amygdalar terminals also received synapses (A, arrow) from elements of inhibitory neurons (A, orange); amygdalar terminals also formed synapses with IDs (B, arrowhead with asterisk on orange shading). (D and E) Amygdalar terminals formed classical triadic arrangements with one ED (green) and one ID (orange). (F, G) Classic triadic arrangements are found in the LGN. Amygdalar terminals in A–E were labeled with the neural tracer FE and visualized with DAB. Yellow asterisks show silver-enhanced gold labeling for PV, and red asterisks in A show TMB rod-like labeling for CB in postsynaptic dendrites. Numbers in B–G indicate distance in nanometers between sections photographed. For color code, see Fig 10. Calibration bar in G applies to B–G. CB, calbindin; DAB, diaminobenzidine; ED, excitatory dendrite; FE, Fluoro- emerald; ID, inhibitory dendrite; LGN, lateral geniculate nucleus; MDmc, mediodorsal thalamic nucleus, magnocellular part; PV, parvalbumin; TMB, tetramethylbenzidine.

All amygdalar boutons in the MDmc also contained multiple mitochondria, classified as RL boutons that form excitatory synapses in the thalamus (Fig 11). Amygdalar boutons formed many puncta adherentia (Fig 11A–11C, arrowheads) and formed synapses preferentially on thalamic thorns (Fig 11A–11C, asterisks and arrowheads). RL boutons generally target proximal dendrites of thalamic relay neurons, in contrast with small excitatory boutons (round vesicles, small terminals [RSs]), which target more distal dendrites, may not contain mitochondria, and are characteristic of most corticothalamic terminals [20,24].

**Fig 12:**
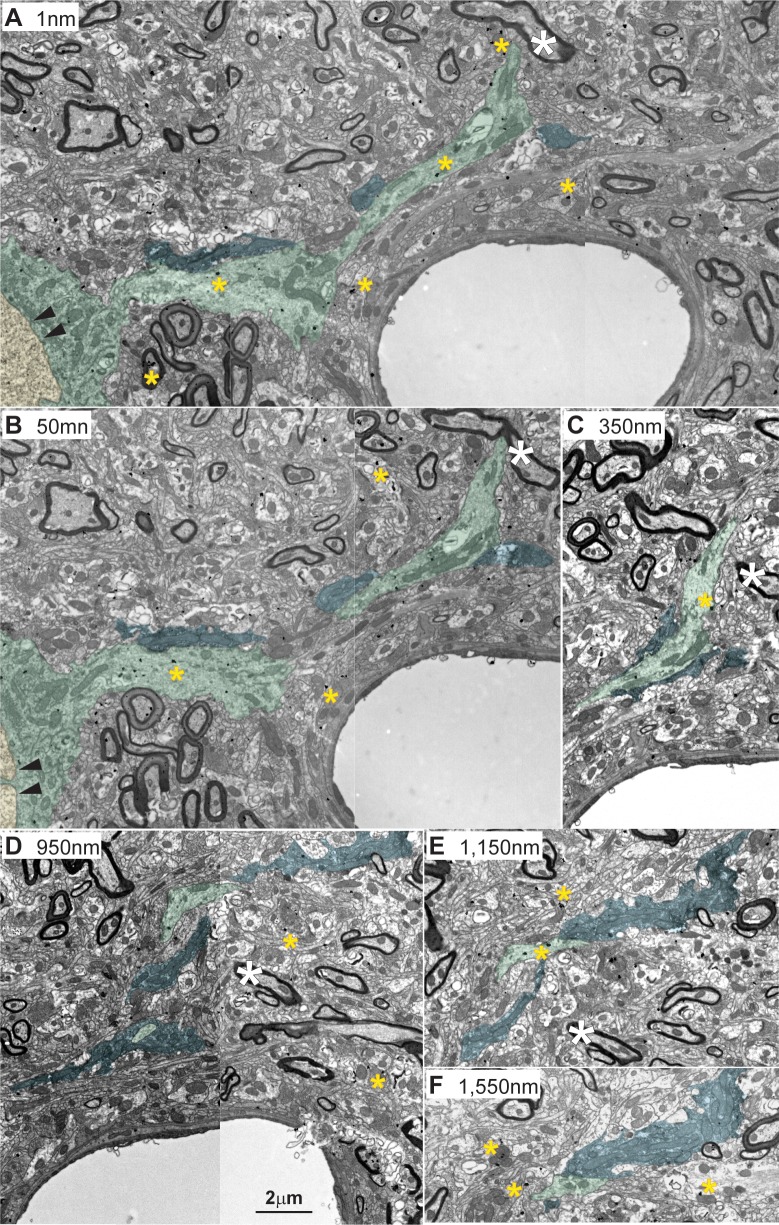
Amygdalar pathways form synapses proximal to neuron cell bodies in the MDmc. (A–F) A series of consecutive sections captured from the electron microscope revealed that amygdala terminals (black label with blue overlay) wrapped around EDs (green) close to the neuron body, identified by the presence of the cellular nucleus (A and B; black double arrowhead and yellow). Amygdalar terminals were labeled with the neural tracer FE and visualized with DAB. Yellow asterisks show silver-enhanced gold labeling for PV in postsynaptic dendrites. Numbers in A–F indicate distance in nanometers between sections photographed. White asterisks show fiduciary marks. Calibration bar in D applies to A–F. DAB, diaminobenzidine; ED, excitatory dendrite; FE, Fluoro-emerald; MDmc, mediodorsal thalamic nucleus, magnocellular part; PV, parvalbumin.

The majority of dendrites of relay neurons expressed one of 2 calcium-binding proteins, with most expressing calbindin (CB) (*n* = 21 of 38; Fig 11, red asterisks), fewer expressing parvalbumin (PV) (*n* = 9; Figs 11 and 12, red asterisks), and the remainder not being labeled within the series (*n* = 8). These calcium-binding proteins describe excitatory PV thalamic “core” pathways known to project to the middle layers from sensory relay nuclei and excitatory CB “matrix” thalamic pathways that project more broadly to the upper layers [26].

### Amygdalar boutons formed synaptic triads in the MDmc

Most amygdalar boutons formed more than one synapse in the MDmc, and half formed synapses with both excitatory and inhibitory neurons (50%, *n* = 19 of 38). Of these boutons, the majority formed synaptic triads that could be identified in the series (74%, *n* = 14 of 19). In this arrangement, IDs were postsynaptic to the excitatory amygdalar bouton and also presynaptic to the same excitatory relay neuron as the amygdalar bouton (Fig 11D and 11E), as described for sensory systems ([27]; Figs 10K, 11F and 11G).

### Synaptic features of amygdalar boutons in the MDmc: 3D analysis and reconstruction

In a more detailed 3D analysis, we followed amygdalar terminations on dendrites through uninterrupted series of sections, ranging from 22 to >220 consecutive sections. We could ascertain that amygdalar axons formed synapses on thick dendrites, characteristic of proximal dendrites, as suggested above by analysis at the level of the system using brightfield microscopy. The proximal amygdalar innervation was attested by 3 relay dendrites that could be traced back to the neuron body (Fig 12).

**Fig 13:**
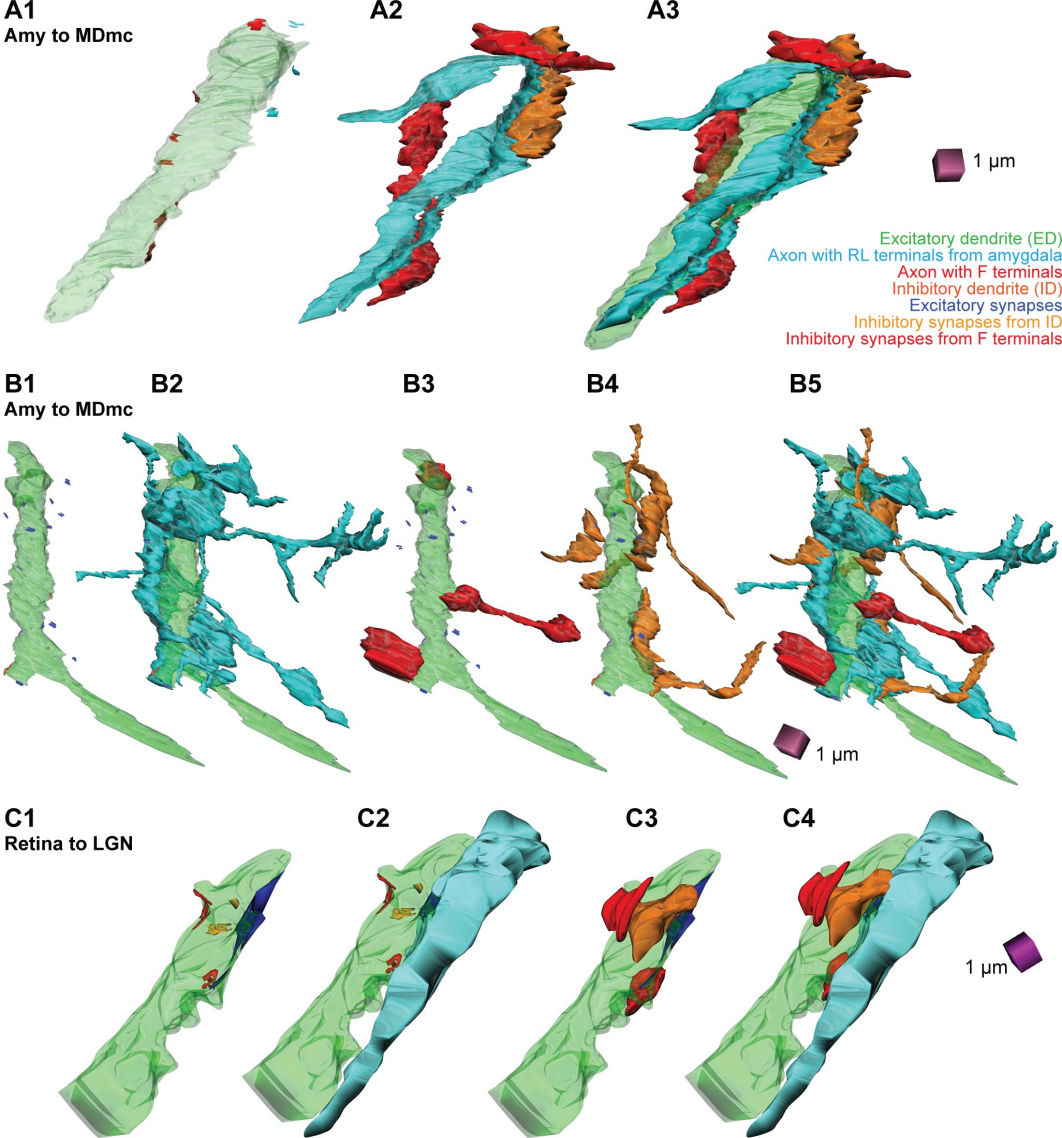
3D reconstruction of amygdalar pathways in the MDmc and comparison with LGN neuropil. (A1) A single ED (green) is shown dotted with excitatory and inhibitory synapses. (A2) Amygdalar terminals (blue) are shown along with their interacting inhibitory axons (red) and IDs (orange). (A3) A1 and A2 are shown together; 3 amygdalar axons (blue) wrap around the relay dendrite (ED, green): one of these axons forms an excitatory synapse with an ED, 2 of the axons form only puncta adherentia with the ED, and one of the latter 2 axons forms 3 synapses with 3 IDs. In this reconstruction, one axon with inhibitory terminals (red) also wraps around the ED and forms 6 inhibitory synapses, and another axon forms one inhibitory synapse with the ED. (B1) An ED (green) is shown alone with synapses by excitatory and inhibitory elements. (B2) The same ED (from B1) is wrapped by amygdalar terminals (blue) with extensive branching; all of these axon terminals are connected and form 4 synapses on the ED, 7 synapses on the IDs, and one triad. (B3) The same ED also contacts 3 inhibitory axons (red). (B4) The ED also contacts 2 IDs (orange). (B5) 3D reconstruction of all elements in B1–B4 shows the complex relations of amygdalar pathways with EDs and inhibitory profiles. (C1–C4) 3D reconstruction of one ED (green) in an LGN neuron targeted by retinal axon terminal (C2, blue) and inhibitory axons (C3 and C4, red) and dendrites (C3 and C4, orange). For color code, see Fig 10. Calibration bar in A3 applies to A1–A3. Calibration bar in B5 applies to B1–B5. Calibration bar in C4 applies to C1–C4. Amy, amygdala; ED, excitatory dendrite; ID, inhibitory dendrite; LGN, lateral geniculate nucleus; MDmc, mediodorsal thalamic nucleus, magnocellular part.

The detailed 3D analysis allowed us to also visualize the complex synaptic arrangements that predominated with large amygdalar boutons, which wrapped around thick relay dendrites (EDs) like vines. These complex arrangements are shown in 3D reconstructions in Fig 13A1–13A3 and Fig 13B1–13B5.

Our analysis included 39 dendrites that were postsynaptic to amygdalar axon boutons (case BL, *n* = 10; case BN, *n* = 29). One or more traced axons enveloped a dendrite with several axon terminals. Most axons formed one or more synapses on the dendrite (*n* = 34 dendrites). Amygdalar axons also formed many puncta adherentia (a nonsynaptic adhesion) on the analyzed relay dendrites. In 5 relay dendrites, we could identify puncta adherentia by amygdalar axons but found no synapses within the series. Other unusual interactions included an axon from the amygdala that formed 4 synapses with 4 inhibitory dendritic segments in the series. Most relay dendrites analyzed also formed synapses with dendrites of inhibitory neurons (IDs; *n* = 36) and with F terminals of inhibitory neurons (*n* = 30).

We could identify classical synaptic triads in 19 of the 39 relay dendrites analyzed, confirming and extending the pattern seen in the 2D analysis (above). The triadic arrangements included amygdalar axons that formed a synapse with a relay dendrite and with one or more IDs, which in turn, formed a synapse on the relay dendrite. In 6 of these relay dendrites, we could identify 2 triads, and in one, there were 3 triads. In 2 of the 39 relay dendrites, we identified a synaptic triad in which 2 inhibitory axon terminals (F) from the same axon formed inhibitory synapses with one ED and one ID; the ID formed one inhibitory synapse with the ED (Fig 10M). Ten of the 39 relay dendrites also formed synapses with RSs.

**Fig 14:**
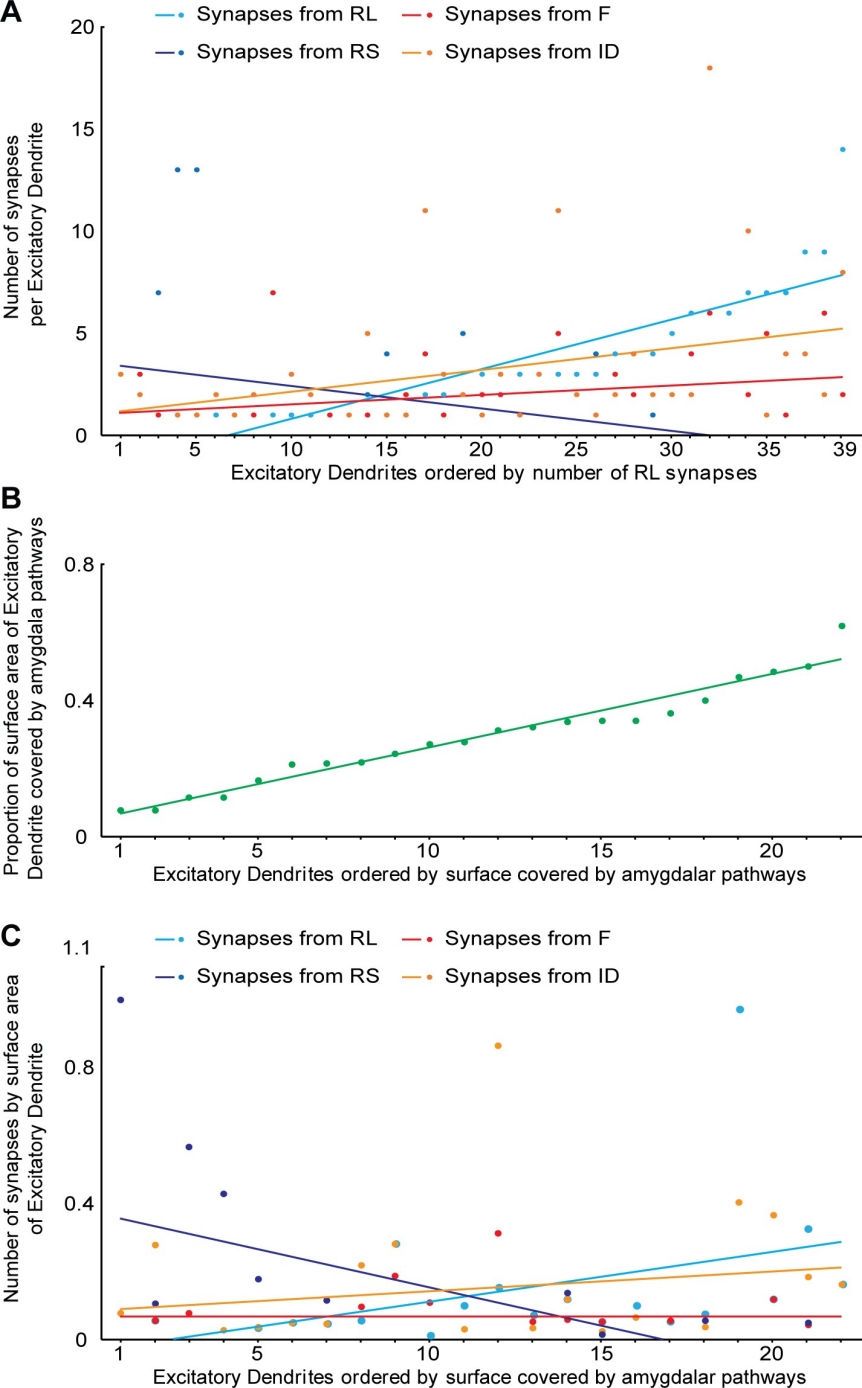
Relationship of EDs that are postsynaptic to amygdalar pathways with presynaptic elements in the MDmc. (A) EDs of the MDmc that are postsynaptic to amygdalar pathways were ordered by the number of RL synapses (light blue) they receive. The number of synapses by RLs is positively correlated with synapses from IDs (orange), shows a small positive correlation with synapses from F terminals (red), and is inversely correlated with the number of synapses from RS terminalss (dark blue). (B) Surface area of EDs covered by the amygdalar pathways ranged from about 10% to over 50% in the series. (C) The density of RS synapses is inversely correlated with the surface of the ED covered by the amygdalar pathway. For color code, see Fig 10. The numerical data underlying this figure can be found in S1 Data. ED, excitatory dendrite; F, inhibitory axon terminal; ID, inhibitory dendrite; MDmc, mediodorsal thalamic nucleus, magnocellular part; RL, round vesicles, large terminal; RS, round vesicles, small terminal.

An analysis of the prevalence of RL boutons that formed synapses is shown for all 39 dendrites in Fig 14A. This analysis revealed that the number of RL synapses (light blue) is inversely correlated with the number of RS synapses (dark blue) and positively correlated with the number of ID synapses (orange) and with F synapses (red). The slope of the large amygdalar boutons (RL) was 3.3 and statistically differed from the slope of RS terminals, which was −1.4 (Fig 1A). We then measured the amount of dendritic surface occupied by the amygdalar pathway in 22 dendrites, which ranged from about 10% to over 50% of the dendrite within our series (Fig 14B). The relationship of the density of synapses by total dendritic surface area covered by amygdalar boutons is shown in Fig 14C. The density of RS synapses is inversely correlated with the surface covered by the amygdalar pathway. This evidence is consistent with our findings that RL synapses are proximal to the cell body, whereas RSs are found at more distal dendritic segments.

The majority of the relay (excitatory) dendrites expressed one of 2 calcium-binding proteins (18 expressed CB, 12 expressed PV, and 9 were not labeled), as also seen in the 2D analysis (above).

### Comparison of amygdalar boutons in the MDmc with excitatory afferents in the LGN

Synaptic triads have been classically described in sensory thalamic nuclei, including retinal afferents that enter the LGN to relay visual signals to the primary visual cortex ([14] reviewed in [15]). We analyzed long electron microscopy (EM) series of sections through LGN that contained 21 representative triads in the neuropil. Each triad included a large retinal axon terminal that formed multiple asymmetric synapses with at least one dendrite of a relay neuron and the dendrite of an inhibitory neuron. The latter dendrite (ID) contained vesicles and, in turn, formed a symmetric (inhibitory) synapse with the same dendrite (ED) of the relay neuron, as shown in Fig 11F and 11G. Other excitatory and inhibitory synapses were also visible on the relay dendrites of LGN neurons. Retinal axons formed large boutons in LGN with large volumes (mean volume ± SE, 74 ± 30 μm^3^, *n* = 21 boutons from one case) and had large diameters (mean diameter ± SE, 3.2 ± 0.26 μm, range 1.9 μm–7.4 μm, *n* = 21 boutons from 2 cases). The mean and range of bouton sizes in LGN were comparable to the sizes seen in our 2D analysis of the amygdalar pathway in the MDmc (above).

The LGN boutons that participated in triads were significantly larger than other excitatory boutons in the LGN neuropil and also contained multiple mitochondria (mean number ± SE, 8.3 ± 0.96) and round vesicles (Fig 11F and 11G). These LGN boutons can be classified as RL boutons, which target proximal dendrites (EDs) of thalamic relay neurons, in comparison with small excitatory boutons with round vesicles (RSs), which target more distal dendrites, may not contain mitochondria, and are characteristic of most corticothalamic terminals [20,24]. Retinal afferents in the LGN and the synaptic triads they formed had similar features as seen between amygdalar afferents in MDmc, including bouton size, presence of mitochondria, and number, type, and target of synapses. However, the synaptic arrangements in the LGN were comparatively simpler (Fig 13C1–13C4) than those in amygdalar pathways in the MDmc, which showed extensive branching (Fig 13B1–13B5).

## Discussion

Our findings reveal that pathways from polymodal and high-order sensory association cortices project to the amygdala at sites that also project to the MDmc. This evidence provides support for sequential processing of signals along these pathways, akin to the processing of sensory signals from the periphery to the thalamus. This conclusion is supported further by similarity of the fine features in the respective pathways and their thalamic organization into triads, highlighting interaction with both excitatory and inhibitory neurons.

### Significance of thalamic synaptic triads

Our comparison of the respective axon boutons in the MDmc and LGN showed remarkable similarity in size and heavy mitochondrial content in these disparate pathways. The parallels extend to classical studies that have described a consistent organization of sensory pathways in the thalamus (e.g., [14]). The visual system is a prime example, whereby retinal pathways provide the first excitatory afferent bouton in the LGN, which forms an asymmetric (excitatory) synapse on a dendrite of a thalamic relay (excitatory) neuron. The same retinal axon bouton forms another synapse on a vesicle-containing dendrite of an inhibitory neuron, which in turn forms an inhibitory synapse on the same relay neuron dendrite, completing the triad [14,15,27,28]. Our findings provide novel, to our knowledge, evidence that the large amygdalar boutons previously described for the mediodorsal thalamic nucleus (MD) in rats and monkeys [19,29] formed synaptic triads that can be compared with the sensory systems, replicated here by neuropil analysis of the LGN.

The functional significance of synaptic triads is not well understood, but their structure suggests a specialized relay mechanism: afferent fibers depolarize both a relay dendrite and a dendrite of an inhibitory neuron, which also forms a synapse with the same relay dendrite. The triadic arrangement allows a short window for initial excitation of a relay neuron followed by inhibition, preventing summation and faithfully transmitting high-frequency signals to the cortex [13]. Our findings suggest that the triadic arrangement of pathways in the thalamus extends beyond the sensory relay nuclei to the high-order MDmc nucleus and may apply to the whole thalamus.

The significance of the triadic arrangement is emphasized further by features of their neurochemistry that affect neuronal response properties. Sensory and other fibers of subcortical origin that project to the thalamus are positive for vesicular glutamate transporter 2 (VGlut2), a specialized glutamatergic axon ending associated with high probability of neurotransmitter release (reviewed in [30]), in a variety of species, including rats and primates (e.g., [31–33]). Similarly, amygdalar fibers that project to the MDmc are positive for VGlut2 and are large [7]. VGlut2 axon terminals are also characteristic of thalamocortical “driver” pathways that innervate the middle cortical layers (e.g., [34]), which effectively activate their postsynaptic targets [35]. Previous studies have emphasized that drivers of high-order thalamic nuclei, such as the MD or the ventral anterior nucleus (VA), originate from large layer 5 cortical neurons (e.g., [22,24,36]; reviewed in [37,38]). Our findings reveal that the subcortical basal amygdala conveys feedforward signals that can effectively activate MDmc neurons as well.

### Why is the amygdala like a sensory structure?

At a global level, the amygdala receives multisensory signals from the entire external environment through late-processing sensory association cortices, shown in part in previous studies and confirmed and extended here (reviewed in [39]). Understanding the sequence of signal processing is more challenging for a complex structure such as the amygdala by comparison with the sensory systems. In the latter, signals can be traced from their origin in the peripheral sensory environment to sensory receptors to the thalamus and then to the primary sensory cortices. By analogy with the sensory cortices, we could ascertain that the amygdala receives feedforward projections that emanate mostly from the upper layers of high-order temporal sensory association cortices, following the same rules as feedforward pathways that connect earlier-processing sensory areas with later-processing sensory areas (reviewed in [39,40]). As in the sensory systems, we found that the same sites in the amygdala that receive sensory-related pathways project to the MDmc, suggesting continuity of pathways.

At the local thalamic level, amygdalar fibers share features with sensory pathways in thalamic relay nuclei by their common organization into synaptic triads. Beyond the similarities, however, the amygdalar pathway displayed a remarkable specialization in the MDmc that distinguished it from sensory thalamic pathways in any species or even from amygdalar axon terminations in the neighboring midline nuclei. The amygdalar pathway targeted strongly only a few neurons in the MDmc, but the innervation was unusually specialized. Strong projections in sensory relay thalamic nuclei have been noted in primate species in the lemniscal and LGN systems (e.g., [41,42]), but none show the complex targeting of single neurons as the amygdala does. Moreover, in the few targeted MDmc neurons, amygdalar axons dominated large dendritic segments by synapses and adhesions, essentially isolating them from other input and dedicating them to the amygdala. The amygdalar terminations in the MDmc also differed from the large and proximal amygdalar terminations in the entirely inhibitory thalamic reticular nucleus [43], which did not similarly encircle the targeted neurons. The specialization of amygdalar endings in the MDmc is likely related to the complexity of signals it receives in comparison with the mapping of the peripheral environment of one modality in sensory relay thalamic nuclei.

### Why is the amygdala like a motor structure?

The amygdala has sensory-related as well as motor-related functions, the latter revealed by its projections to hypothalamic and brainstem autonomic structures that are activated during emotional arousal (reviewed in [4]). The complex motor-related functions of the amygdala can also be understood by its intricate projection to the MDmc, as seen here. The MD, in general, receives strong projections from the basal ganglia [6,44], including the ventral pallidum that projects to the MDmc [29]. Perhaps the best indicator of the motor-related function of the amygdala is by analogy with another sequential pathway from the superior colliculus to the lateral part of the MD, which projects to the frontal eye field (FEF). In lateral MD, burst activity after collicular stimulation was considered to be a corollary discharge, a predictive signal sent from the effector collicular system to convey to the FEF the trajectory of an impending saccade [45]. Burst activity in the thalamus can amplify and facilitate transmission to cortex (e.g., [46]), and integrity of MD was critical for accuracy and precision of predictive saccades in behaving monkeys [45,47]. In addition, the MD selects actions based on evaluation of rewards [48], which may be transmitted from the amygdala along the pathway described here.

The classical theory of corollary discharges is traced to Helmholtz [49] and later elaborated by Sperry ([50]; discussion in [51]). The idea of corollary discharges in the nervous system was first proposed to explain how the brain may distinguish between self-initiated and other movements. In the case of eye movements, corollary discharges serve to cancel the illusion of movement of the environment as the eyes move and images shift on the retina. It has since become apparent that corollary discharges are more general in the brain and help monitor cognitive operations as well [52]. Burst activity—associated with fast and faithful transmission of signals—has similarly been recorded in the primate amygdala in association with the coding of salient signals [53]. Neurons in the macaque monkey amygdala fire during social engagements and, significantly, can distinguish decisions taken by the self or by social partners [54,55]. The amygdala is in a strategic position to evaluate the entire external and internal environment based on the current status of goals and drives and to convey corollary signals to activate MDmc neurons, which then project to the prefrontal cortex. Our findings thus suggest extension of the employ of corollary discharges from motor effectors in the brain to a system associated with affective significance and updating of rewards for action. The ultimate test of this hypothesis lies in the realm of functional studies.

### Integrative sensory-motor functions of the amygdala, MDmc, and pOFC and disruption in schizophrenia

**Fig 15:**
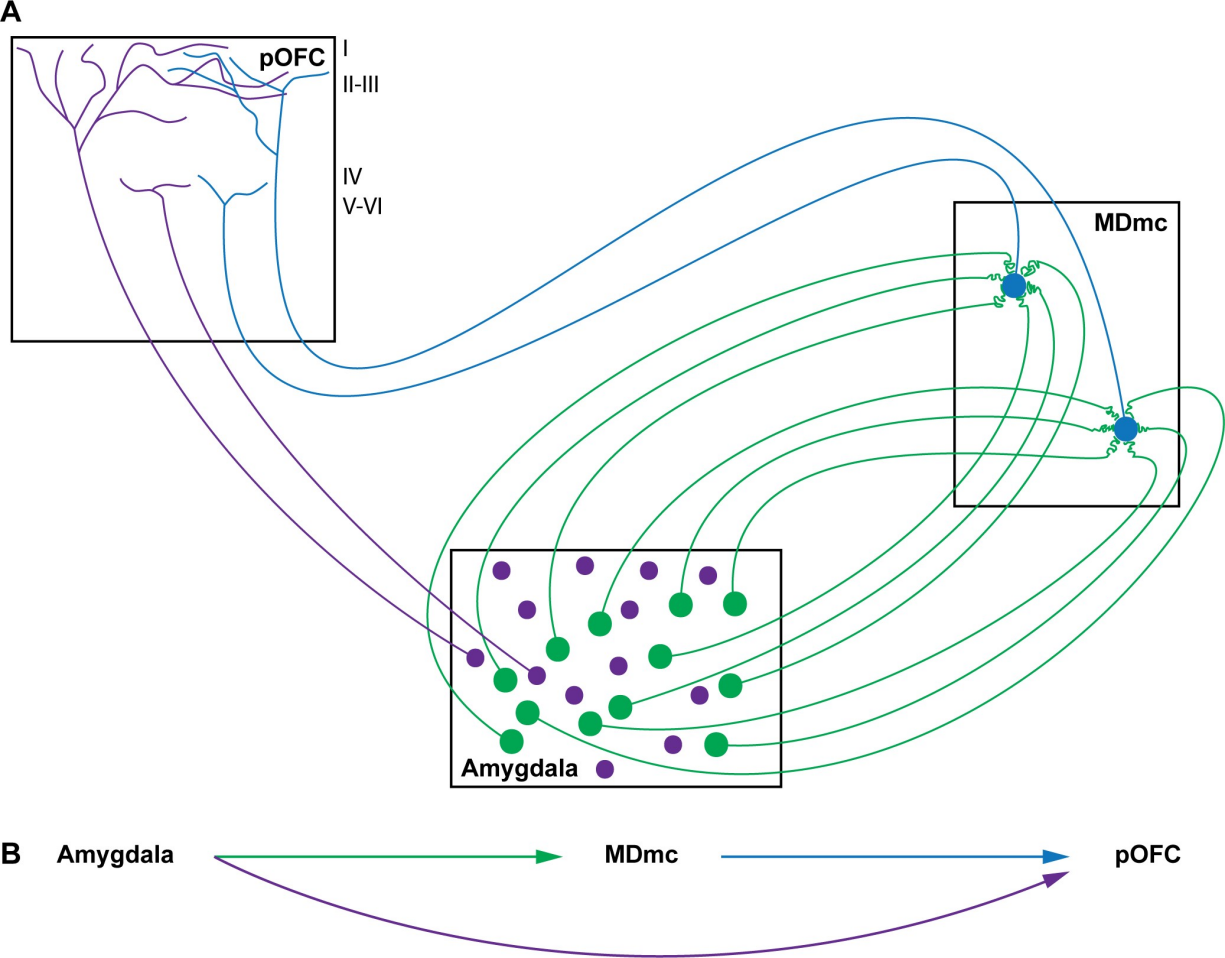
Summary of projections from the amygdala. (A) Hypothetical focused projection from the amygdala to distinct MDmc neurons (green) and diffuse projection to the pOFC (purple). (B) Summary of amygdalar pathways to the MDmc (green arrow) and from the MDmc to the pOFC (blue arrow), and direct projection from the amygdala to the pOFC (purple arrow). Roman numerals indicate cortical layers. MDmc, mediodorsal thalamic nucleus, magnocellular part; pOFC, posterior orbitofrontal cortex.

The functions of the MD and the amygdala must also be viewed in the context of their major connections with the prefrontal cortex, which uses a variety of signals for decision and action (reviewed in [39,56]). The major cortical target of the MDmc is the pOFC. The amygdala and pOFC receive in common input from the same high-order sensory association areas and from limbic areas concerned with the internal milieu and can be considered environmental integrators (reviewed in [39]). In addition to the thalamic route, the amygdala projects directly to pOFC [17,57] in a pathway that differs markedly from the pathway to MDmc (Fig 15). The direct amygdalar pathway to the pOFC originates from smaller neurons that are positive for vesicular glutamate transporter 1 (VGlut1) [7], which is associated with modulatory pathways. However, what the amygdalar pathway to the pOFC lacks in strength in individual synapses may be amply made up by its massive projection [58].

The question arises about the possible function of the direct amygdalar pathway to the pOFC and the indirect pathway through the thalamic MDmc. The multimodal input to the amygdala [reviewed in [39]] suggests that the direct pathway to the pOFC may convey information about the status of the external (sensory) and internal (emotional) environment. On the other hand, the projection from the amygdala to the MDmc and then to the pOFC may be related to motor functions. This hypothesis is based on physiologic and behavioral findings on the interaction of the MD and the amygdala with the prefrontal cortex for updating information during sequential tasks (e.g., [59,60]; reviewed in [37]). Updating desired goals and predicted outcomes is likely conveyed from the amygdala to the thalamic MDmc as a corollary discharge for fast transmission to pOFC. Coordinated activation of the direct and indirect amygdalar pathways to pOFC thus appears to be essential for comparing the status of the entire environment to predict the outcome for decision and action.

The interactions of pathways that underlie the complex processes for decision and action are disrupted in psychosis (reviewed in [61]) and in anxiety disorders that affect emotional processing through the amygdala (e.g., [5]). Considerable evidence suggests disruption of corollary discharge mechanisms in schizophrenia, affecting the sense of agency and attribution of thoughts, desires, and actions to external sources (e.g., [62–70]). The thalamocortical motif that links the thalamus with high-order association prefrontal cortices is likely vulnerable to disruption along multiple dimensions, including the known deficits in eye movements in schizophrenia, as shown in computational studies [71]. Our findings suggest that deficits in the highly specialized amygdalar pathway in the MDmc, which projects to the pOFC, may disrupt the coordination of this thalamic pathway with a direct pathway from the amygdala to the pOFC (Fig 15). A defect in this integrated tripartite system of the amygdala, MDmc, and pOFC may disrupt the timing of signals that convey the status of the external and internal emotional environments to the orbitofrontal cortex and perturb the prediction of outcomes for decision and action in schizophrenia.

## Materials and methods

### Ethics statement

Experiments were conducted according to the Guide for the Care and Use of Laboratory Animals [72]. Experimental methods were approved by the Institutional Animal Care and Use Committee at Boston University School of Medicine (#AN-15157), Harvard Medical School, and New England Primate Research Center (#01303). Procedures involving animals were designed to reduce the number of animals needed and minimize animal suffering.

### Experimental design

The experimental design is based on placement of bidirectional tracers in the amygdala of rhesus monkeys to label pathways linking the amygdala with the cerebral cortex and the thalamus (Fig 1A–1C). Our first goal was to investigate cortical pathways from sensory cortices to the amygdala (Fig 1A and 1B). We addressed this issue by tracing these pathways retrogradely after injections in the amygdala. Our second goal was to map pathways from the amygdala to the thalamus (Fig 1B and 1C). We addressed this issue by tracing these pathways anterogradely and retrogradely after injections in the amygdala at the level of the system and the synapse using light microscopy and EM. We also provide an overview of the synaptic circuitry in the primate thalamus to facilitate understanding of synaptic circuits linking the amygdala and thalamus and compare the synaptic arrangements of amygdalar pathways with retinal pathways to the thalamus. Data of the type of analysis performed on each animal are summarized in Table 1.

### Animal surgery, tracer injection, and brain cutting

Experiments were conducted on 5 rhesus monkeys (*Macaca mulatta*) aged 2–3.5 years of both sexes. One of these cases (BN) was also used to study the neuropil in the LGN (Table 1).

Prior to surgery, we imaged the brain using magnetic resonance imaging following animal sedation with ketamine hydrochloride and propofol anesthesia in order to calculate stereotaxic coordinates for the injection sites. Experiments were conducted under sterile conditions, and animals were continuously monitored for respiratory rate, oxygen saturation, heart rate, and temperature. For surgery, we placed the animals under isoflurane anesthesia, positioned them in a stereotaxic apparatus (Kopf 1430M, David Kopf Instruments; Tujunga, CA, USA), and made a small opening in the skull and dura. We injected 10% dilutions of Fluoro-emerald (FE, fluorescein dextran, 3 μl, mixture of 3 kDa and 10 kDa; Invitrogen Cat#A889; RRID: AB_221561; D3306; D1820; Carlsbad, CA, USA), Fluoro-ruby (FR, tetramethylrhodamine dextran, 3–4 μL, mixture of 3 kDa and 10kDa; Invitrogen Cat #A6397; RRID: AB_1502299 D3308; D1817), or biotinylated dextran amine (BDA, 6 μl, mixture of 3 kDa and 10 kDa; Cat #D7135; D1956; Invitrogen) into amygdalar nuclei of the right hemisphere using Hamilton syringes (10 μl; Reno, NV, USA). Dextran amines (FE, FR, and BDA) of 10 kDa molecular mass are optimal for anterograde tracing and label the entire extent of axon terminals. Dextran amines (FE, FR, and BDA) of 3 kDa also act as retrograde tracers and label cell bodies and proximal dendrites of projection neurons [73–75]. Each tracer was injected in 2–4 penetrations; the needle was left in place for 10–15 minutes to allow tracer penetration at the injection site and prevent upward diffusion of the dye during retraction of the needle.

After 18–21 days, the animals were given an overdose of sodium pentobarbital and perfused transcardially with 4% paraformaldehyde, 0.2% glutaraldehyde in 0.1 M phosphate-buffered saline (PBS [pH 7.4]). The brain was removed, cryoprotected in ascending sucrose solutions (10% to 25% sucrose %wt/vol in 0.1 M PBS [pH 7.4] with 0.05% sodium azide; Sigma-Aldrich, St. Louis, MO, USA), frozen in isopentane (Thermo Fisher Scientific; Pittsburgh, PA, USA), at −80°C, and cut on a freezing microtome (AO Scientific Instruments, Reichert Technologies; Buffalo, NY, USA), in 50 μm coronal sections to form 10 series. We stored sections free floating at −20°C in a solution of 30% ethylene glycol, 30% glycerol, 0.05% sodium azide in 0.05 M phosphate buffer (pH 7.4).

### Tissue processing to view amygdalar terminations in the MDmc: Brightfield microscopy

Tissue sections of one series (1 in 20 sections) were processed to visualize amygdalar terminations in the thalamus. To view amygdalar pathways labeled with BDA tracer, free-floating sections were rinsed with PBS (0.01 M [pH 7.4]). Tissue sections were then incubated at 4°C with glycine (0.05 M in PBS, Sigma-Aldrich) for 1 hour, hydrogen peroxide (0.3% in PBS, Sigma-Aldrich) for 30 minutes, and in preblocking solution for 1 hour (10% normal goat serum [NGS, Vector Laboratories Cat# S-1000, RRID:AB_2336615, Burlingame, CA, USA], 10% bovine serum albumin [BSA, Sigma-Aldrich], 0.2% BSA-c [Aurion, Wageningen, The Netherlands], and 0.2% Triton X-100 [Sigma-Aldrich] in 0.01 M PBS). After the preblock, tissue sections were incubated in avidin-biotin horseradish peroxidase (AB-HRP; AB-Kit, Vector Cat# PK-6100, RRID: AB_2336827) at a 1:100 dilution in PBS for 1 hour and then processed with diaminobenzidine (DAB substrate kit; Vector Cat# SK-4100, RRID: AB_2336382) for 2–3 minutes.

For cases with FE and FR injections, tissue sections went through glycine incubation and hydrogen peroxide incubation, as above. We then incubated tissue sections with avidin-biotin blocking solutions (AB block, Vector Cat# SP-2001, RRID: AB_2336231) followed by preblocking solution and incubated at 4°C for 2 days with primary antibodies to tracers (rabbit anti-FE, Invitrogen Cat# A889, RRID: AB_221561; rabbit anti-FR, Invitrogen Cat# A6397, RRID: AB_1502299) at 1:800 in preblocking solution. During incubation, we microwaved the tissue sections twice a day (3 minutes on, 2 minutes off, 3 minutes on, at 150 W in a Biowave; Ted Pella, Redding, CA, USA) to enhance antibody penetration. After PBS rinsing, we incubated tissue sections overnight in secondary antibody solution (biotinylated goat anti-rabbit IgG, Vector Cat# BA-1000, RRID: AB_2313606) at 1:200 in preblocking solution at 4°C with 2 microwave sessions, followed by AB-HRP incubation and DAB, as described above. After every step, we rinsed tissue sections with PBS (3 × 10 minutes, 0.01 M [pH 7.4]).

To study the cytoarchitecture of amygdalar terminations in the thalamus, we counterstained with Nissl some of the sections that were also processed to view pathways. Briefly, air-dried sections were rinsed with 0.1 M PB (pH 7.4) and placed in 1:1 chloroform-ethanol solution for 3 hours, rehydrated in descending ethanol solutions (100%, 95%, 70%), washed in dH_2_O, stained with 0.05% thionin for 1–2 minutes, washed in dH_2_O, dehydrated with series of ascending ethanol solutions (70%, 95%, 100%), cleared in xylene, and coverslipped with mounting medium (Entellan, Merck, Whitehouse, NJ, USA).

### EM: Immunohistochemistry

To study pathways in the electron microscope, we used triple immunohistochemistry to identify tracers with DAB (which appears as uniform dark precipitate under EM) and calcium-binding proteins (CB and PV) using gold labeling with silver enhancement (forms clumps of round particles) and tetramethylbenzidine (TMB) staining (forms rod-shaped precipitate) as described [76]. Tissue sections were incubated as above in 0.01 M sodium citrate (pH 8.5) for 30 minutes at 35°C and 0.05 M glycine for 1 hour at 4°C, and any BDA tracer was blocked with AB blocking solutions (AB block, Vector Cat# SP-2001, RRID: AB_2336231). Background binding was blocked with incubation for 1 hour at 4°C in 5% NGS, 5% BSA, 0.025% Triton X-100 (Roche Applied Sciences; Indianapolis, IN, USA), 0.1% acetylated BSA-c (Aurion), and 3.5% mouse blocking reagent (MOM basic kit, Vector Cat# MKB-2213, RRID: AB_2336587) in PBS. Sections were then bound overnight at 4°C with antibodies for tracers (FE or FR: 1:800 in 1% NGS, 1% BSA, 0.1% BSA-c, 0.025% Triton X-100, and 8% MOM protein concentrate [MOM basic kit, Vector] in PBS; rabbit polyclonal IgG anti-FE, Invitrogen Cat# A889, RRID: AB_221561; rabbit polyclonal IgG anti-FR, Invitrogen Cat# A6397, RRID: AB_1502299) and one of 2 calcium-binding proteins (CB or PV: 1:2000, mouse monoclonal IgG anti-CB, Swiss Antibodies Cat #300, RRID: AB_10000; mouse monoclonal IgG anti-PV, Swiss Antidodies Cat #235, RRID: AB_10000343, Bellizona, Switzerland). All primary and secondary antibody incubations included an 8-minute microwave run as above.

Sections were rinsed in PBS, then incubated for 6 hours at 25°C with biotinylated secondary antibodies for tracers (biotinylated goat anti-rabbit IgG, Vector Cat# BA-1000, RRID: AB_2313606; 1:200 in 1% NGS, 1% BSA, 0.1% BSA-c, 0.025% Triton X-100, 8% mouse blocking reagent [MOM basic kit, Vector], and 0.1% cold water fish gelatin [Aurion] in PBS) and gold-conjugated secondary antibodies for CB and PV (1:50 UltraSmall ImmunoGold F[ab] fragment of goat anti-mouse IgG, Aurion Cat #800.266, RRID: AB_2315632). Sections were then postfixed with 3% glutaraldehyde and 1% paraformaldehyde in PB in a microwave oven (Biowave, Ted Pella; 2 minutes at 150 W, 4°C). Sections were rinsed in glycine (5 minutes) and rinsed in PB (2 × 10 minutes), followed by enhancement conditioning solution (ECS, Aurion; 1:10, 2 × 10 minutes). Gold-conjugated proteins were visualized by silver enhancement for 60–90 minutes (R-Gent SE-EM, Aurion); the tissue was then rinsed in ECS (2 × 10 minutes) and then PB (2 × 10 minutes). Tracers were visualized with DAB, as above. For all rinses following silver enhancement, we used 0.1 M PB (pH 7.4), and in some pieces of tissue, the order of labeling was reversed to control for any attraction between gold and biotin. Any remaining biotin binding sites were blocked with AB blocking solutions, and then any remaining mouse binding sites were blocked with incubation for 1 hour at 4°C in 3.5% mouse blocking reagent (MOM basic kit, Vector) in 5% NGS, 5% BSA, 0.025% Triton X-100, and 0.1% BSA-c in PB.

We incubated sections overnight at 4°C with antibody for a second calcium-binding protein (mouse monoclonal IgG anti CB or PV, Swiss Antibodies: 1:2,000 in 1% NGS, 1% BSA, 0.1% BSA-c, 0.025% Triton X-100, and 8% mouse blocking reagent [MOM basic kit, Vector] in PB), followed by rinses in PB and incubation for 1–2 hours at 25°C in biotinylated secondary antibody (biotinylated goat anti-mouse IgG, Vector, Cat #BA-9200, RRID: AB_2336171; 1:200 in 1% NGS, 1% BSA, 0.1% BSA-c, 0.025% Triton X-100, and 8% mouse blocking reagent [MOM basic kit, Vector] in PB), then rinsed in PB and incubated in AB-HRP as above. These calcium-binding proteins were visualized with TMB staining as follows: sections were first incubated for 15 minutes in 0.005% TMB (Sigma-Aldrich), 0.004% ammonium chloride (Sigma-Aldrich), and 5% ammonium paratungstate (Sigma-Aldrich) in 0.1 M PB (pH 6.0) and then incubated for 1–5 minutes in the same solution plus 0.005% hydrogen peroxide (Sigma-Aldrich) until staining appeared. The staining was stabilized by incubating sections for 10 minutes in a solution of 0.05% DAB (SigmaFast DAB tablet, Sigma-Aldrich), 0.02% cobalt chloride (Sigma-Aldrich), 0.004% ammonium chloride (Sigma-Aldrich), and 0.005% hydrogen peroxide (Sigma-Aldrich) in 0.1 M PB (pH 6.0). Finally, sections were rinsed in PB and postfixed in 6% glutaraldehyde and 2% paraformaldehyde in PB with a microwave oven (Biowave, Ted Pella; 150 W at 15°C) until sample temperature reached 30–35°C [77]. We conducted control experiments on tissue by omitting primary antibodies and incubating in secondary antibody solutions, and no immunolabeling was observed.

### EM: Embedding and serial sectioning

Sections were rinsed in PB (3 × 20 minutes) and postfixed for 15 minutes in 1% osmium tetroxide (Electron Microscopy Sciences, Hatfield, PA, USA) with 1.5% potassium ferrocyanide (Electron Microscopy Sciences) in PB with a microwave oven (Biowave, Ted Pella; 100 W at 12°C, 2 minutes on, 2 minutes off, 2 minutes on) and rinsed in PB (3 × 2 minutes) and water (3 × 2 minutes). Sections were then rinsed in 50% ethanol (3 × 5 minutes), stained with 1% uranyl acetate (30 minutes in 70% ethanol; Electron Microscopy Sciences), and dehydrated in a series of ethanols (90%, 95%, 100%; 3 × 5 minutes each). For embedding, sections were infiltrated with propylene oxide (2 × 7 minutes; Electron Microscopy Sciences), and then a 1:1 mixture of araldite (Electron Microscopy Sciences) and propylene oxide (1 hour). Sections were infiltrated with araldite overnight, followed by flat embedding in araldite in aclar (Ted Pella). Aclar-embedded tissue was cured for 48 hours at 60°C. Small pieces of tissue 500- to 750-μm wide were cut from each section and re-embedded in araldite blocks and cured for 48 hours at 60°C. To reconstruct postsynaptic sites, araldite blocks containing embedded tissue from the MDmc were sectioned at 50 nm using an ultramicrotome (Leica Ultracut UCT, Leica Microsystems; Buffalo Grove, IL, USA) and collected on pioloform-coated copper slot grids to form a series of approximately 20 to >200 sections.

Fixed tissue sections at the level of the LGN with no immunohistochemical staining were processed for EM following the same protocol, as above. The goal was to identify typical retinothalamic synaptic triads [12].

### Mapping of labeled axons and neurons

We studied labeled pathways from the amygdala to temporal cortices and the thalamus in sections stained for tracers (BDA, FE, and FR). Some of the sections processed for pathway tracing were counterstained to place areal, laminar, and nuclear boundaries as described previously for temporal areas [11] and for thalamic nuclei [78]. We studied the distribution of retrogradely labeled neurons from the amygdala in temporal areas under brightfield and darkfield illumination (Olympus optical microscope, BX 60 and BX 51; Tokyo, Japan) to identify laminar patterns of cortical pathways to the amygdala. We identified amygdala pathways to the thalamus under brightfield and darkfield illumination (Olympus optical microscope, BX 60 and BX 51), and we plotted clusters of labeled axon terminals in the MDmc with the aid of a semiautomated commercial system (StereoInvestigator; MicroBrightField, Williston, VT, USA).

### Axon terminal population analysis

To describe the presynaptic features of amygdala pathways to the thalamus, we acquired image stacks of several focal planes at 1,000× to study the size of boutons from the amygdala in the MDmc and the midline thalamic nuclei. The size of boutons is correlated with synaptic efficacy [79–81]. We used ImageJ (NIH, Bethesda, MD, USA) to combine image stacks into a composite image with high depth of field focused throughout the *z*-axis as described [82]. Labeled boutons from amygdala pathways were traced manually using the open software program Reconstruct (http://www.bu.edu/neural/Reconstruct.html; [83]), and data were exported to a database in Excel. More than 5,000 boutons in the MDmc and 9,000 boutons in midline thalamic nuclei were measured for major diameter. We used k-means cluster analysis of the major bouton diameter to determine a cutoff point and separate labeled boutons into groups of large and small boutons.

### EM: Data analysis and 3D reconstruction

Using 80-kV transmission EM at 16,000×–26,000× (JEOL 100CX; Jeol, Peabody, MA, USA), we exhaustively sampled several sections to identify approximately 10–30 labeled boutons in each series. We viewed labeled and unlabeled axon terminals and dendrites participating in synaptic arrangements in MD and LGN at high magnification (10,000× to 30,000×). We captured high-resolution images with a digital camera (ES1000W; Gatan, Pleasanton, CA, USA) attached to the electron microscope (100CX; Jeol). We photographed boutons throughout their entire extent (between 20 and > 200 serial ultrathin sections) and imported images as a series in Reconstruct (http://www.bu.edu/neural/Reconstruct.html; [83]), in which we aligned sections through coregistration of corresponding fiducial marks (e.g., mitochondria) between adjacent sections. We then traced boutons and other pre- and postsynaptic structures (e.g., dendrites). We calibrated section thickness through measurements of the diameter of mitochondria as described previously [81]. We reconstructed structures in 3D and calculated their volumes, surface areas, and diameters. We used estimated volumes and diameters from the 3D EM analyses for regressions and correlations. We used classic criteria to identify triads, synapses, and profiles as described previously [12,27,43,81]. We prepared photomicrographs using Adobe Photoshop (RRID: SCR_014199) and Adobe Illustrator (RRID: SCR_014198; Adobe Systems, San Jose, CA) and adjusted overall brightness and contrast without retouching.

### Photography

We photographed labeled boutons, axons, and neurons in cortical areas of the temporal lobe and in thalamic nuclei under brightfield and darkfield illumination using an optical microscope (Olympus BX 51) with a CCD camera (Olympus DP70) connected to a personal computer with a commercial imaging system (DP Controller).

At the EM, labeled boutons from the amygdala and their postsynaptic profiles were captured using a digital camera (ES1000W, Gatan) at a magnification of 10,000×–30,000×. Images were imported into Adobe Illustrator CC software (Adobe Systems) to assemble in figures. Minor adjustment of overall brightness and contrast were made, but images were not retouched.

## Supporting information

S1 Data (XLSX)

## Abbreviations

AB-HRP: avidin-biotin horseradish peroxidase
BDA: biotinylated dextran amine
BL: basolateral nucleus
BM: basomedial nucleus
BSA: bovine serum albumin
Ca: anterior commissure
CB: calbindin
Cd: caudate nucleus
Ce: central nucleus
Cim: central intermediate nucleus
Cl: claustrum
CnMd: centromedian nucleus
Co: cortical nucleus
Csl: central superior lateral nucleus
DAB: diaminobenzidine
DC: dense-core vesicle
ED: excitatory dendrite
EM: electron microscopy
F: inhibitory axon terminal
FD: fascia dentata
FE: Fluoro-emerald
FEF: frontal eye field
FR: Fluoro-ruby
ID: inhibitory dendrite
La: lateral nucleus
LD: lateral dorsal nucleus
LGN: lateral geniculate nucleus
MD: mediodorsal thalamic nucleus
MDmc: mediodorsal thalamic nucleus, magnocellular part
MDpc: mediodorsal thalamic nucleus, parvicellular part
Me: medial nucleus
NGS: normal goat serum
Pa: paraventricular nucleus
PBS: phosphate-buffered saline
Pcn: paracentral nucleus
Pf: parafascicular nucleus
pOFC: posterior orbitofrontal cortex
Pt: paratenial nucleus
PV: parvalbumin
R: thalamic reticular nucleus
Re: nucleus reuniens
RL: round vesicles, large terminal
RS: round vesicles, small terminal
Sm: stria medullaris
Thi: habenulointerpeduncular tract
TMB: tetramethylbenzidine
VA: ventral anterior nucleus
VGlut1: vesicular glutamate transporter 1
VGlut2: vesicular glutamate transporter 2
VLc: ventral lateral nucleus caudal part
VPI: ventral posterior inferior nucleus
VPLc: venteral posterior lateral nucleus caudal part
VPM: ventral posterior medial nucleus
VPMpc: VPM, parvicellular part
WM: white matter
